# An H2A Histone Isotype, H2ac, Associates with Telomere and Maintains Telomere Integrity

**DOI:** 10.1371/journal.pone.0156378

**Published:** 2016-05-26

**Authors:** Chia-Hsin Su, Ching Cheng, Tsai-Yu Tzeng, I-Hsuan Lin, Ming-Ta Hsu

**Affiliations:** 1 Institute of Biochemistry and Molecular Biology, School of Life Science, National Yang-Ming University, Taipei, 11221, Taiwan, Republic of China; 2 VYM Genome Research Center, National Yang-Ming University, University System of Taiwan, Taipei, 11221, Taiwan, Republic of China; 3 Chien-Tien Hsu Cancer Research Foundation, Taipei, 11221, Taiwan, Republic of China; Texas A & M University, UNITED STATES

## Abstract

Telomeres are capped at the ends of eukaryotic chromosomes and are composed of TTAGGG repeats bound to the shelterin complex. Here we report that a replication-dependent histone H2A isotype, H2ac, was associated with telomeres in human cells and co-immunoprecipitates with telomere repeat factor 2 (TRF2) and protection of telomeres protein 1 (POT1), whereas other histone H2A isotypes and mutations of H2ac did not bind to telomeres or these two proteins. The amino terminal basic domain of TRF2 was necessary for the association with H2ac and for the recruitment of H2ac to telomeres. Depletion of H2ac led to loss of telomeric repeat sequences, the appearance of dysfunctional telomeres, and chromosomal instability, including chromosomal breaks and anaphase bridges, as well as accumulation of telomere-associated DNA damage factors in H2ac depleted cells. Additionally, knockdown of H2ac elicits an ATM-dependent DNA damage response at telomeres and depletion of XPF protects telomeres against H2ac-deficiency-induced G-strand overhangs loss and DNA damage response, and prevents chromosomal instability. These findings suggest that the H2A isotype, H2ac, plays an essential role in maintaining telomere functional integrity.

## Introduction

The telomere is a specialized chromatin structure at the end of a chromosome for protecting the termini of eukaryotic linear chromosomes from degradation, end-to-end fusion and undesired recombination. In general, telomeres consist of a 6–8 base-pair sequence with variable numbers of repeats in different species. The actual terminus of a telomere consists of a 3' protrusion of the G-rich single-strand overhang [1,2]. The human G-rich strand overhang is believed to loop back to invade and hybridize to the double-stranded hexamer (TTAGGG/CCCTAA) repeats as the t-loop [3]. This special structure has also been observed in different species using electron microscopy [3], demonstrating that it is an evolutionally conserved and essential feature of telomeres. Although at present, it is not clear whether t-loops are present at all chromosome ends, or whether nucleosomes or histones are required for constructing the t-loops, studies in human and mouse cells suggest that the consequence of chromosome fusions has been identified as the result of degradation of the G strand [4,5]. This indicates that the specialized chromatin structure is required for protecting the chromosome ends.

In human, telomeres comprise 5–15 kb of double-stranded TTAGGG/CCCTAA repeat sequences that end with a ~50–300 nucleotide single-stranded G-rich sequence (the G-rich overhang) [1,2]. Human telomeric chromatin contains a set of telomeric DNA binding proteins, including the fission yeast telomeric DNA-binding protein Taz1 orthologs, TTAGGG-repeat factor-1 (TRF1) and TRF2 [6,7], as well as the orthologs of protection of telomeres protein 1, POT1. TRF1 and TRF2 contain a similar C-terminal Myb domain that mediates sequence-specific binding to telomeric DNA; however, TRF2 is different from TRF1 in that its N terminus is very basic rather than acidic [7,8]. TRF1 is reported to be involved mainly in the control of telomere length, and TRF2 is mainly implicated in chromosome end protection, by preventing end-to-end fusions [9,10]. POT1 was originally discovered in *Schizosaccharomyces pombe* [11], and POT1 has subsequently been identified in a wide range of eukaryotes, including plants and human, thus is highly conserved from yeast to mammals [11]. All POT1 homologs contain two highly conserved oligonucleotide binding (OB) folds that have high affinity to bind the G-rich single strand overhang [11,12]. TRF1 and TRF2 directly bind to double-stranded telomeric DNA, and the connection between TRF1 and TRF2 by TIN2 (TRF1-interacting factor-2) contributes to the stabilization of TRF2 on telomere [13]. TRF2 also recruits hRAP1, a homolog of yeast RAP1 protein [14], to human telomeres. In contrast to TRF1 and TRF2, POT1 binds to the 3’ G-rich overhang sequences through its OB folds [12]. In addition, the interaction of TPP1 (POT1 binding partner)-TIN2 regulates the bridging between TRF1-TRF2 and POT1 and promotes as well as stabilizes the assembly of high-order telomeric complexes named the telosome or shelterin complex [13,15]. Studies of cells and mice that are deficient in the individual proteins of the shelterin complex supports a model in which telomere dysfunction, owing either to the loss of telomeric repeats or causing genome instability results from the loss of the telomere protective structure.

In addition to the specific telomeric complex, human telomeres are organized in heterochromatin-like structures and are accompanied by histones of trimethylation of H3K9 and H4K20 [16–18] that have the ability to silence subtelomeric genes through telomere position effect [19]. Human telomeres and subtelomeres are both characterized by a high content of DNA repeats, and subtelomeres have similarity with pericentromeric regions that are gene-poor, whereas telomeres do not contain genes at all. Nevertheless, unlike yeast, in which only subtelomeric repeats contain nucleosomes [20], both human telomeres and subtelomeres contain nucleosomes [21,22]. Moreover, diffuse micrococcal nuclease digestion patterns reveals that human telomeres and subtelomeres display a bipartite structure with an unusual chromatin structure that had a shorter repeat size than bulk nucleosome spacing, suggesting a special spacing of nucleosomes at the telomere and an extensive array of canonical chromatin structure in the proximal part of telomere [21,22]. However, whether these unusual nucleosomes contain canonical histones or whether these histones carry specific modifications are not known and further analysis would be needed to decipher the detailed structure of the telomere as well as subtelomere chromatin structures. Recently, histone modifications at the telomere have been shown to participate in the regulation of telomere functions. SIRT6-mediated H3K9 deacetylation is required for the stable association of WRN at telomere chromatin [23] and a dynamic H4K12 acetylation is involved in the regulation of telomere-related processes, including telomere replication, transcription and recombination [24].

Despite the great efforts spent in elucidating telomere structure and function, it is still far from a clear how telomeres are organized, especially in higher eukaryotes. Moreover, it should be noted that most research has not taken into account the nature of the histones that directly interact with telomeric sequences or other telomeric complexes. Whether histones and specific telomeric proteins cooperate in forming the telomeric complex or whether histone directly participates in the regulation of telomere structure or function has not been characterized.

In the human genome there are sixty-four replication-dependent histone genes with different isotypes associated with each class of the five histone types H1, H2A, H2B, H3 and H4 [25]. The biological meaning of the redundant isotypes is not well understood. In a previous report, we showed that one of the isotypes of H2A, H2ac, serves as a master regulator of estrogen receptor alpha (ERα)-dependent gene activation through the recruitment of ERα and mediates chromatin looping of regulatory elements of estrogen receptor-targeted genes [26]. In this report, we further showed that H2ac was specifically associated with the telomere through the interaction with the amino-terminal domain of TRF2. We also discovered that H2ac depletion induced the rapid loss of telomere repeat sequences, loss of G-strand overhangs and resulted in telomere dysfunction. These dysfunctional telomeres became associated with DNA damage response factors, such as 53BP1, phospho-γ-H2AX Ser139, phospho-ATM Ser1981, and XPF nuclease. Overall, our results indicate that H2ac plays an essential role in the maintenance of telomere length and the protection of chromosome ends from XPF nuclease.

## Results

### Association of a histone H2A isotype, H2ac, with telomeric sequences

**Fig 1:**
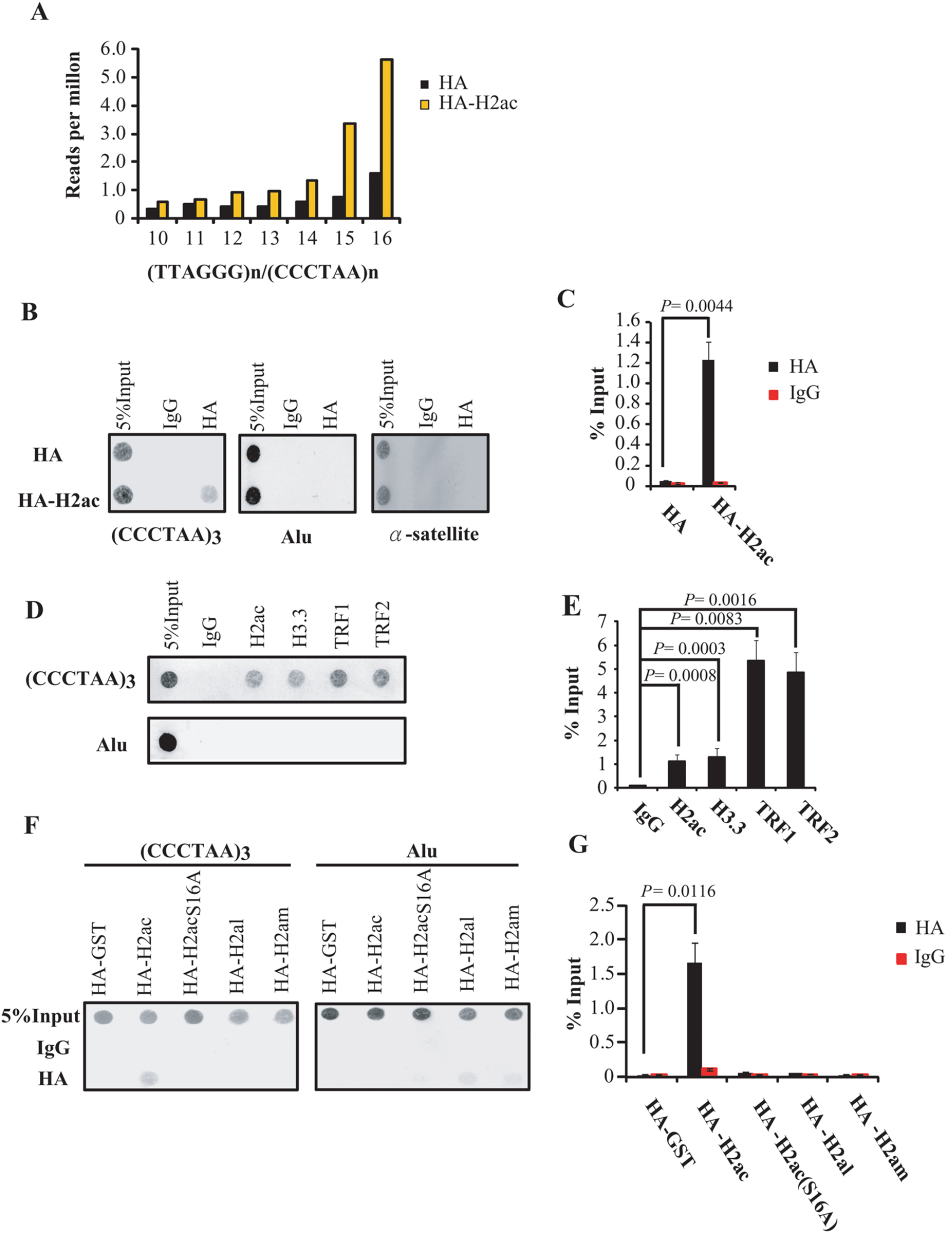
H2ac associates with telomeres. (A) Distribution of perfect TTAGGG_n_ /CCCTAA_n_ repeats in the ChIP-seq data performed on cells overexpressing HA or HA-H2ac. Control HA ChIP is shown in black and HA-H2ac in yellow. (B) Dot-blot hybridization to analyze association of telomeric DNA repeat with HA-H2ac. The indicated cells were fixed by formaldehyde and were subjected to ChIP using anti-HA antibodies followed by dot blot analysis using a (CCCTAA)_3_ probe, Alu sequence and α-satellite. (C) The signals of telomeric DNA in ChIP were quantified and normalized to the corresponding total telomeric signal (Input). The *P* value was calculated using a Student's two-tailed *t*-test. (D-E) Dot-blot hybridization to analyze the association of telomeric DNA repeats with endogenous H2ac. The association of telomeric DNA repeat with endogenous histone H3.3, TRF1 and TRF2 served as the positive controls. The signals of telomeric DNA in ChIP were quantified and normalized to the corresponding total telomeric signal (Input). The *P* value was calculated using a Student's two-tailed *t*-test. (F-G) Analysis of specificity of interaction between H2ac and telomeric sequence using HA tagged GST, H2ac mutant, and H2al and H2am overexpressed in MCF-7 cells. The signals of telomeric DNA in ChIP were quantified and normalized to the corresponding total telomeric signal (Input). The *P* value was calculated using a Student's two-tailed *t*-test.

We previously showed that an H2A isotype, H2ac, regulated ERα through the binding to the estrogen receptor and through H2ac-mediated chromatin looping of regulatory elements of estrogen receptor-targeted genes [26]. During subsequent analysis of H2ac binding sites in the genome using hemagglutinin (HA)-tagged H2ac by genome-wide chromatin immnuoprecipitation (ChIP)-seq in MCF-7 cells, we found that the 100-bp-long raw reads were enriched with TTAGGG_15-16_ telomeric repeat sequences in HA-H2ac ChIP-seq data as compared with those in the HA control (Fig 1A). This observation suggests the binding of H2ac to telomeres. To validate the ChIP-seq result, we performed a telomere-ChIP assay [27] to examine the association of HA-H2ac with telomeric repeat DNA using anti-HA antibody to immunoprecipitate protein-associated DNA fragments followed by dot blot hybridization analysis using a digoxigenin (DIG)-labeled telomere-specific probes. We showed that DNA immunoprecipitated with anti-HA antibody was enriched for telomeric DNA in HA-H2ac-overexpressed cells as compared with HA-overexpressed cells (Fig 1B and 1C). We further performed telomere-ChIP assays using anti-H2ac antibody to confirm endogenous H2ac association with the telomere; the association of histone H3.3, TRF1 and TRF2 with telomeres [15,28] served as positive controls (Fig 1D and 1E). Additionally, no hybridization signals were observed in the two additional H2ac knockdown cell lines (S1 Fig). These results suggest that H2ac is associated with the telomere repeat sequence.

To examine the specificity of the association of H2ac with the telomeric sequence, we performed telomere-ChIP experiments using cells transfected with HA-GST, as well as with mutation the S16A of HA-H2ac, which was shown previously as a critical element in controlling ERα activity [26]. No telomeric DNA was immunoprecipitated in these ChIP experiments (Fig 1F and 1G). We also performed telomere-ChIP in cells overexpressing two other H2A subtypes, HA-H2al and HA-H2am. No hybridization signals were observed in these ChIP experiments (Fig 1F and 1G) indicating that binding of the telomere is specific to the H2ac isotype.

### H2ac binds telomeric proteins TRF2 and POT1 but not TRF1, TIN2 or TPP1

**Fig 2:**
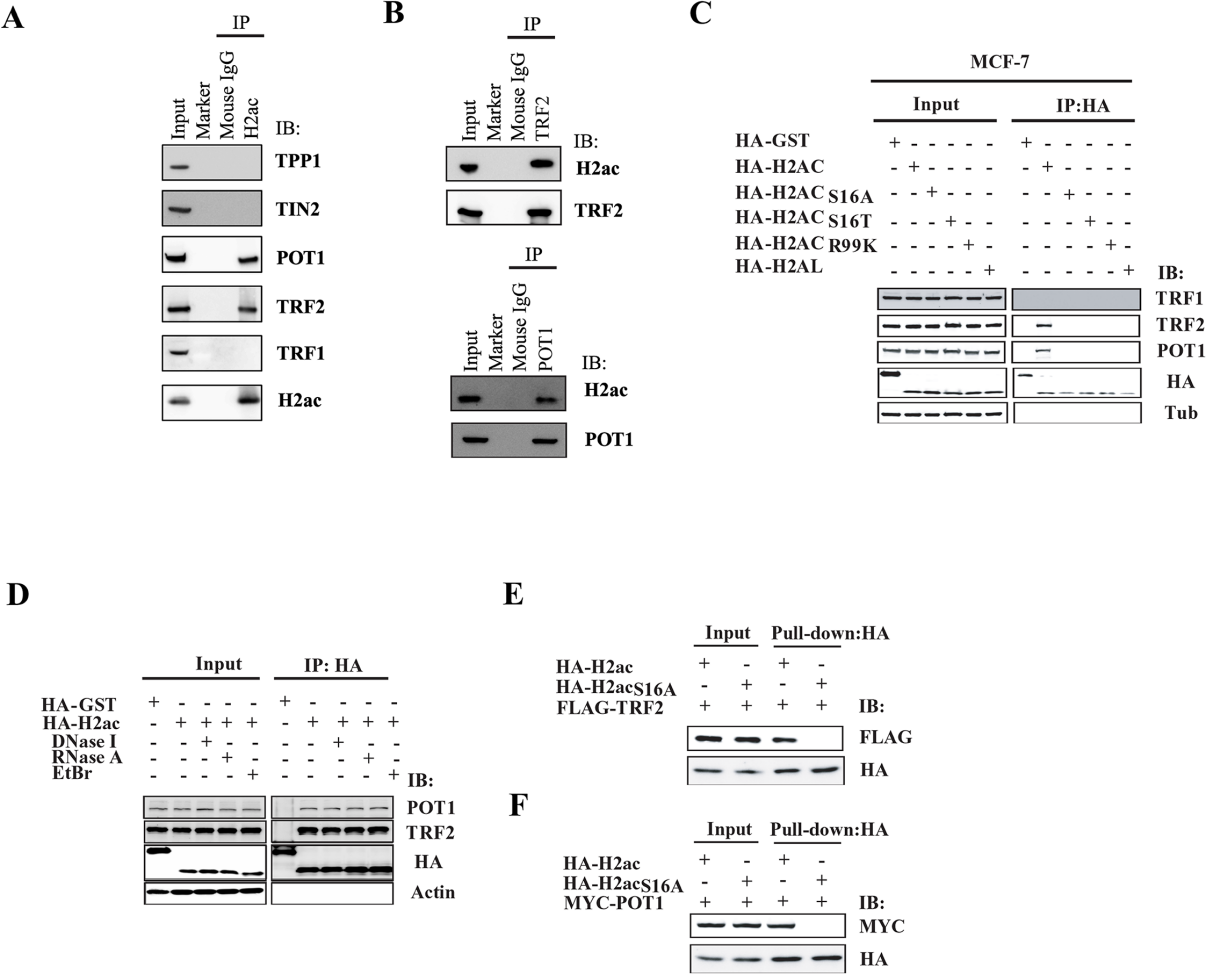
Analysis of the interaction of H2ac with TRF2 and POT1 by co-immunoprecipitation. (A) Endogenous H2ac interacts with endogenous TRF2 and POT1 in MCF-7 cells. Nuclear extracts were immunoprecipitated with mouse anti-H2ac and were blotted with antibodies against TPP1, -POT1, -TRF2, -TIN2, -TRF1 and -H2ac antibodies. Mouse IgG was used as a negative control. (B) Co-immunoprecipitation assay with anti-TRF2 (top) or anti-POT1 (bottom) and western blot with anti-H2ac in MCF-7 nuclear extracts. (C) The wild-type or mutant HA-H2ac was transiently expressed in MCF-7 cells. MCF-7 nuclear lysates were immunoprecipitated with anti-HA antibody, followed by immunoblotting with anti-TRF1, anti-TRF2 and anti-POT1 antibodies, respectively. (D) Association of H2ac-TRF2 and H2ac-POT1, respectively in MCF-7 cells were not affected after treatment with DNase I, EtBr or RNase A. (E) Purified recombinant HA-H2ac interacted directly with purified recombinant FLAG-TRF2. HA-H2ac_S16A_ was used as a negative control. (F) Purified recombinant HA-H2ac interacted directly with purified recombinant MYC-POT1. HA-H2ac_S16A_ was used as a negative control.

Given that mammalian telomeres are capped and protected by the telosome/shelterin complex which consists of three telomeric DNA binding factors (TRF1, TRF2 and POT1), as well as TPP1 and TIN2 [3,12,13,15], we investigated whether endogenous H2ac interacted with these factors. We performed co-immunoprecipitation using mouse anti-H2ac antibody from the nuclear extract of MCF-7 followed by western blot analysis using antibodies against the five shelterin complex proteins. The results indicated strong association of TRF2 and POT1 with H2ac, whereas no associations were detected between H2ac and TRF1, TIN2 and TPP1 (Fig 2A). The interaction between H2ac and TRF2/POT1 was also observed by reciprocal co-immunoprecipitation (Fig 2B). Specificity of the antibodies was confirmed by competitive western blotting analysis with H2ac, TRF2 and POT1 proteins (S2 Fig).

To demonstrate the specific association of H2ac with TRF2 and POT1, we overexpressed HA-H2ac followed by western blot analysis using antibodies against TRF1, TRF2 and POT1. The results indicated that TRF2 and POT1 co-immunoprecipitated with HA-H2ac, whereas no association was detected between HA-H2ac and TRF1 (Fig 2C). A control experiment using HA-GST did not bring down TRF2 or POT1. Overexpression of HA-tagged H2ac mutants (S16A, S16T and R99K) and another H2A histone isotype, H2al, did not result in the immunoprecipitation of TRF2 and POT1 (Fig 2C), indicating specificity of H2ac in binding to these factors.

To examine whether the association of H2ac with TRF2 or POT1 in the nuclear extract is DNA-dependent, we repeated the co-immunoprecipitation experiments in the presence of DNase I or ethidium bromide (EtBr) using anti-HA antibody and probed with anti-TRF2 or anti-POT1 antibody. H2ac-TRF2 or H2ac-POT1 interactions were observed after treatment with DNase I or EtBr indicating that DNA tethering was not responsible for the associations of H2ac-TRF2 or H2ac-POT1 (Fig 2D). Recently, it was reported that telomeric repeat-containing RNA (TERRA) is part of the telomeric complex [29]. Therefore, we further tested whether TERRA was necessary for H2ac to interact with TRF2 or POT1. Coimmunoprecipitation after RNase A treatment did not affect H2ac-TRF2 or H2ac-POT1 interactions (Fig 2D). Therefore, it is also unlikely that H2ac binds to TRF2 or POT1 through DNA or RNA.

To exclude the possibility that H2ac associates indirectly with TRF2 and POT1 through the involvement of other proteins, we synthesized HA-tagged H2ac, FLAG-tagged TRF2 and MYC-tagged POT1 by an *in vitro* transcription—translation coupled reaction using plasmid clones containing the three genes and analyzed their association *in vitro* by pull-down assay. Consistent with the *in vivo* interaction assays, HA-tagged H2ac readily brought down FLAG-tagged TRF2, but not by the HA-tagged H2ac mutant control (Fig 2E). Similarly, the association of HA-tagged H2ac and MYC-tagged POT1 was detected by anti-MYC but not in the H2ac mutant control (Fig 2F). These *in vitro* protein association studies indicate unequivocally that H2ac interacts directly with TRF2 and POT1.

### The amino terminal basic domain of TRF2 is required for H2ac recruitment at telomeres

**Fig 3:**
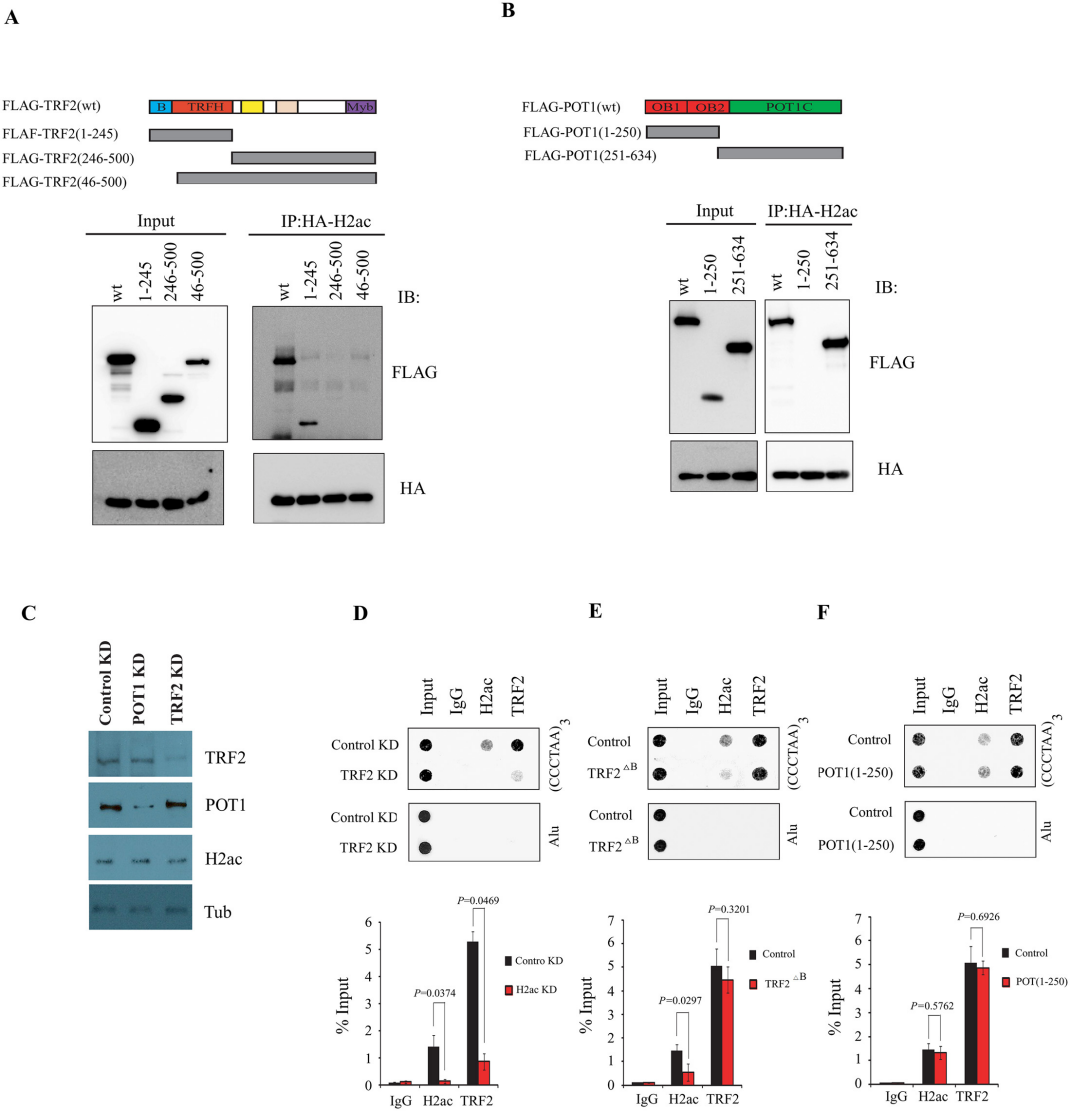
The amino-terminal basic domain of TRF2 is required for H2ac recruitment to telomeres. (A) Co-expression of HA-H2ac and truncated mutants of TRF2 were fused to FLAG and blotted with anti-FLAG in MCF-7 nuclear extracts. (B) Co-expression of truncated mutants of POT1 were fused to FLAG and blotted with anti-FLAG in MCF-7 nuclear extracts. (C) Western blotting of cell extracts prepared from control KD, POT1 KD and TRF2 KD cells using antibodies labeled on the right. (D) siControl or siTRF2-transfected MCF-7cells were analyzed by ChIP assay with anti-TRF2, anti-H2ac, or IgG control and were probed by hybridization with (CCCTAA)_3_ probe or Alu sequences. Quantification of telomeric-repeat DNA recovered in each ChIP is shown. (E) TRF2^△B^ (TRF2_46-500_) transfected MCF-7 cells or control cells were assayed by ChIP with antibodies to TRF2, H2ac, or control IgG and probed with (CCCTAA)_3_ probe and Alu sequences. Quantification of telomeric-repeat DNA recovered in each ChIP is shown. (F) POT1(1–250) transfected MCF-7 cells or control cells were assayed by ChIP with antibodies to TRF2, H2ac, or control IgG and were probed with (CCCTAA)_3_ probe and Alu sequences. Quantification of telomeric-repeat DNA recovered in each ChIP is shown.

To investigate the domain of TRF2/POT1 that is important for its interaction with H2ac, we co-expressed HA-H2ac with FLAG-TRF2(wt), FLAG-TRF2(1–245), FLAG-TRF2(246–500) or FLAG-TRF2(46–500) (FLAG-TRF2^ΔB^) lacking the basic domain (Fig 3A). Co-immunoprecipitation with anti-HA antibody showed that only FLAG-TRF2(wt) and FLAG-TRF2(1–245) were able to interact with HA-H2ac (Fig 3A). These results suggest that the N-terminal basic domain of TRF2 is required and is sufficient for TRF2 interaction with H2ac. We also identified a subdomain of POT1 responsible for H2ac recruitment by co-expressing HA-H2ac with FLAG-POT1(wt), FLAG- tagged OB1 with partial OB2 domain of POT1 (1–250) as well as FLAG-tagged C-terminal protein interaction domain (251–634) of POT1 in MCF-7 cells. Western blotting analysis showed that the C-terminal fragment of POT1 is responsible for the interaction with H2ac (Fig 3B).

To further determine whether TRF2 was required for H2ac recruitment to telomeres, we tested the effect of TRF2 knockdown on the association of H2ac with telomere-repeat DNA in MCF-7 cells. TRF2 was efficiently depleted in MCF-7 cells transfected with siTRF2 but not in cells transfected with control siRNA (Fig 3C). siTRF2 had no detectable effect on overall H2ac protein levels in transient transfection assays 72 hr after transfection. By using telomere-ChIP assays, we found that TRF2 depletion dramatically led to a decrease in the association of H2ac with telomere repeat DNA (Fig 3D). Similarly, overexpression of TRF2 deletion mutant TRF2^ΔB^ resulted in a significant decreased in the association of H2ac with telomere-repeat DNA by telomere-ChIP assay (Fig 3E). We also found that overexpression of POT1_1-250_ had no effect on H2ac association with telomeric DNA (Fig 3F). These findings support the model that the amino-terminal basic domain of TRF2 is required for H2ac recruitment to telomeres.

### Telomere-repeat loss and dysfunction in H2ac depleted cells

**Fig 4:**
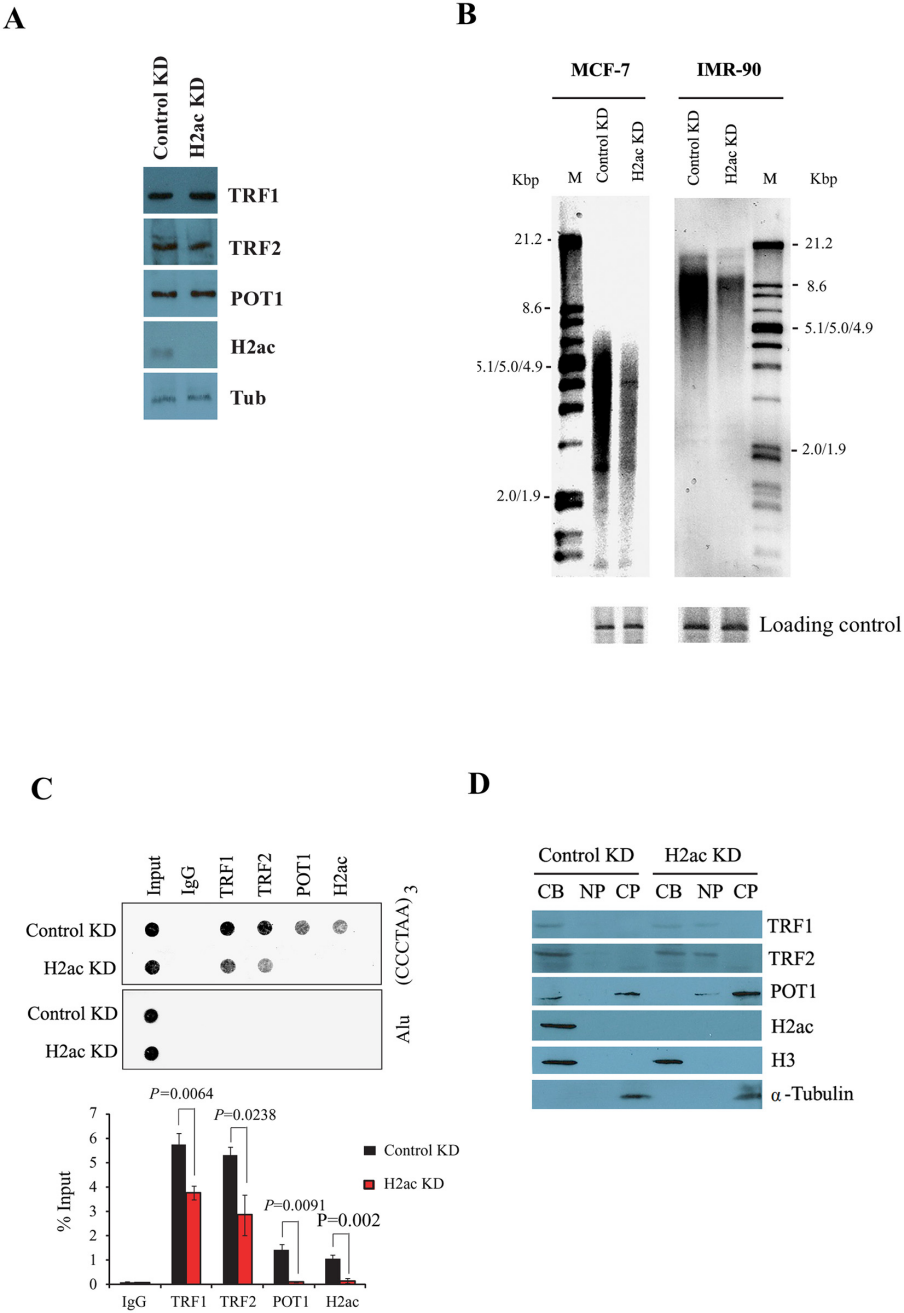
H2ac depletion induces telomere-repeat loss and telomere dysfunction. (A) Western blotting of cell extracts prepared from control KD and H2ac KD cells using antibodies labeled on the right. (B) Telomere length analysis of MCF-7 and IMR-90 cells with control or H2ac siRNAs by telomere restriction fragment (TRF) analysis. MCF-7 and IMR-90 cells were harvested at day 5 after two separate transfections with control and H2ac siRNA. Telomere-repeat length and intensity was measured by restriction digest of genomic DNA with AluI/MboI and Southern hybridization with DIG-labeled (TTAGGG)_4_ probe (top panel). The GAPDH region was used as a control for DNA loading (bottom panel). The position of molecular weights (MWs; kb) is indicated on the left. (C) Telomere-ChIP assay indicates the effect of H2ac depletion on the occupancy of TRF1, TRF2 and POT1 at telomeres with telomere-specific sequences or Alu sequences using dot blotting. Quantification of TTAGGG repeat DNA in the panel recovered in each ChIP is shown. The average of experiments performed in triplicate is shown. The *P* value was calculated using a Student's two-tailed *t*-test. (D) Effect of H2ac depletion on soluble and chromatin-bound TRF1, TRF2 and POT1 proteins. Equal cell equivalents of cytoplasmic proteins (CP), nucleoplasmic proteins (NP), and the chromatin-bound fraction (CB) were analyzed. α-tubulin is cytoplasmic and histone H3 is a chromatin protein.

Wang *et al*. showed that overexpression of TRF2^ΔB^ led to a loss of telomere-repeat signal intensity in restriction fragment-length assays [30]. We reasoned that if H2ac was a functionally relevant target of the amino-terminal basic domain of TRF2, H2ac depletion should have a similar effect as overexpression of TRF2^ΔB^. To test this possibility, we first analyzed the effect of H2ac knockdown on telomere DNA quantity using telomere restriction fragment (TRF) analysis [31]. MCF-7 and IMR-90 cells were harvested at day five after transfections with H2ac siRNAs. Knockdown of H2ac did not alter the overall expression of TRF1, TRF2 or POT1 at the protein level (Fig 4A). The telomere length and quantity were measured by restriction digest of genomic DNA with AluI/MboI and Southern hybridization with a DIG-labeled (TTAGGG)_4_ probe. As shown in Fig 4B, depletion of H2ac resulted in greatly reduced telomeric DNA signals in both MCF-7 and IMR-90 cells but not with control siRNA. The GAPDH gene served as loading control (Fig 4B). We also designed two scrambled sequences of H2ac specific siRNA to examine the specificity of H2ac siRNA. The data showed that expression of H2ac and telomeric DNA signals were not altered in MCF-7 with two H2ac scrambled siRNAs relative to the control. In contrast, expression of H2ac and the signal of telomeric DNA repeats were dramatically decreased in MCF-7 with H2ac specific siRNA (S3 Fig). Furthermore, depletion of other H2A isotypes, H2al and H2am, did not result in the loss of telomeric DNA signal indicating that the effect is not due to general alteration of cell physiology by the depletion of H2A-type histone but is specific to the H2ac isotype (S4 Fig). These data indicate that H2ac depletion leads to loss of telomere repeat DNA.

Because H2ac depletion caused rapid loss of telomere repeat sequences, we examined the effect of H2ac knockdown on the association of TRF1, TRF2 and POT1 with telomere by the telomere-ChIP. Using antibodies against TRF1, TRF2 and POT1, we found that association of these proteins with telomeres was reduced after H2ac knockdown (Fig 4C). Knockdown of H2ac did not alter the overall expression of TRF1, TRF2 and POT1 at the protein level (Fig 4A), thus excluding the interpretation that a decrease in the association of these factors with telomeres is due to inhibition of the synthesis of these factors.

To further confirm this observation, chromatin, nucleoplasmic and cytoplasmic fractions of MCF-7 cells were prepared for western blotting. Depletion of H2ac resulted in a decrease in the chromatin-bound signal of TRF1, TRF2 and POT1 (Fig 4D). The marker proteins for the cytoplasmic fraction (α-tubulin) and the chromatin fraction (histone H3) were consistent with their respective localizations. The data suggest that H2ac is required for maintenance of telomere integrity to prevent the loss of telomeric repeat sequences.

### H2ac is required for genome stability

**Fig 5:**
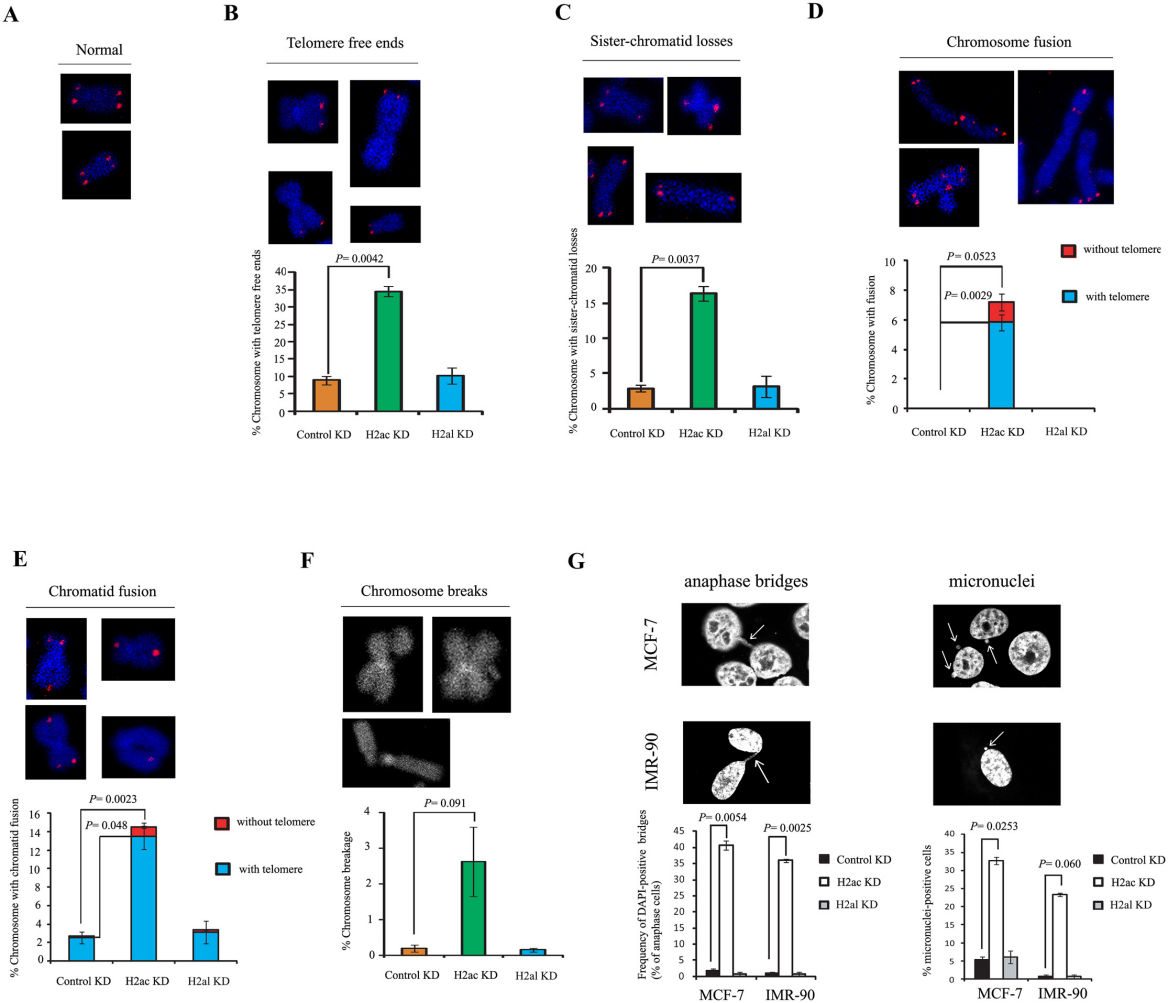
H2ac depletion induces chromosomal abnormalities. Telomeric FISH on metaphases spreads from MCF-7 cells with siH2ac. The telomeric hybridization signal is shown in red and DAPI-counterstained chromosomes in blue. (A) Representative images of normal telomeric DNA signals (in red) and chromosomes (in blue). (B-F) Representative images of multiple chromosome abnormalities. Knockdown of H2ac induced multiple chromosomal aberrations, including chromosome with telomere free ends (B); sister-chromatid telomere losses (C); chromosome fusions with or without TTAGGG repeats at the fusion sites (D); chromatid fusions (E); and chromosome breakages (F). The frequency of cytogenetic aberrations is quantitated under each panel. (G) The induction of anaphase bridges and micronuclei is shown. MCF-7 and IMR-90 cells were harvested at day 6 after two separate transfections with control or H2ac siRNAs. The black-and-white images showed DNA staining by DAPI. Quantification of DAPI-positive anaphase bridges and micronuclei in control or H2ac siRNA-treated in MCF-7 or IMR-90 cells. (n = 100, the *P* value was calculated using a Student's two-tailed *t*-test.).

Telomere dysfunction induced by TRF2 inhibition can initiate genomic instability [10,30]. van Steensel *et al*. showed that chromosomes in cells expressing TRF2^△B△M^ exhibited abnormally high occurrences of mitotic defects, including lagging chromosomes, anaphase bridges, and associations between chromosome ends [10]. To explore whether H2ac is involved in genome stability, we examined chromosomal abnormalities by fluorescence *in situ* hybridization of telomere (telomere-FISH) in metaphase chromosomes from MCF-7 cells expressing H2ac-siRNA for six days. Consistent with the TRF assay, we also found significantly high frequencies of chromosomes without telomeric signals or with greatly reduced telomeric signals (34.4% of chromosome ends lacked telomeric signals and 16.3% of sister-chromatid telomere losses) in H2ac-depleted cells (Fig 5B and 5C). Given that overexpression of TRF2^△B^ also increased telomere signal-free ends [30], our results suggest that H2ac might cooperate with TRF2 in maintaining telomere repeats.

Cytological examination revealed that there was a significant increase in chromosome abnormalities in H2ac knockdown cells as compared with control knockdown or with knockdown using another H2A isotype, H2al. In H2ac knockdown MCF7 cells, end-to-end chromosome fusions with (5.82%) or without (1.38%) telomeres were observed in 7.2% of all chromosomes examined (Fig 5D), and 14.51% of sister chromatin fusions with (13.53%) or without (0.98%) telomere signals (Fig 5E) as well as 2.6% of chromosome breakages (Fig 5F) were observed in H2ac-depleted cells. These cytological abnormalities were rarely observed in the control siRNA knockdowns or knockdown using H2al siRNA. These results suggest that H2ac is required for telomere maintenance and genome stability and the cytological abnormalities observed are not due to histone depletion *per se*.

In addition, cytological analysis revealed the frequent occurrence of anaphase bridges in H2ac knockdown cells (Fig 5G). This phenotype was rarely observed in cells treated with control siRNA and H2al-siRNA. The incidence of anaphase bridges was quantitated in a total of 100 anaphase cells expressing H2ac-siRNA, control siRNA and H2al-siRNA. At day 6 after depletion of H2ac, 42% of the MCF-7 cells and 35% of IMR-90 cells had one or more anaphase bridges (Fig 5G). In contrast, the level of anaphase bridges was below 2% in the control and H2al knockdown cells (Fig 5G). Cytological analysis of H2ac-depleted cells also revealed the frequent occurrence of micronuclei in interphase cells (Fig 5G). In addition, telomere hybridization signal and γ-H2AX pS139 were observed in micronuclei (S5 Fig). These data suggest that telomere-damaged cells caused by H2ac knockdown exhibited mitosis with unresolved telomere repair and increased occurrence of micronuclei and other genomic aberrations.

### H2ac silencing-induced ATM-dependent DNA damage response pathway and cell senescence

**Fig 6:**
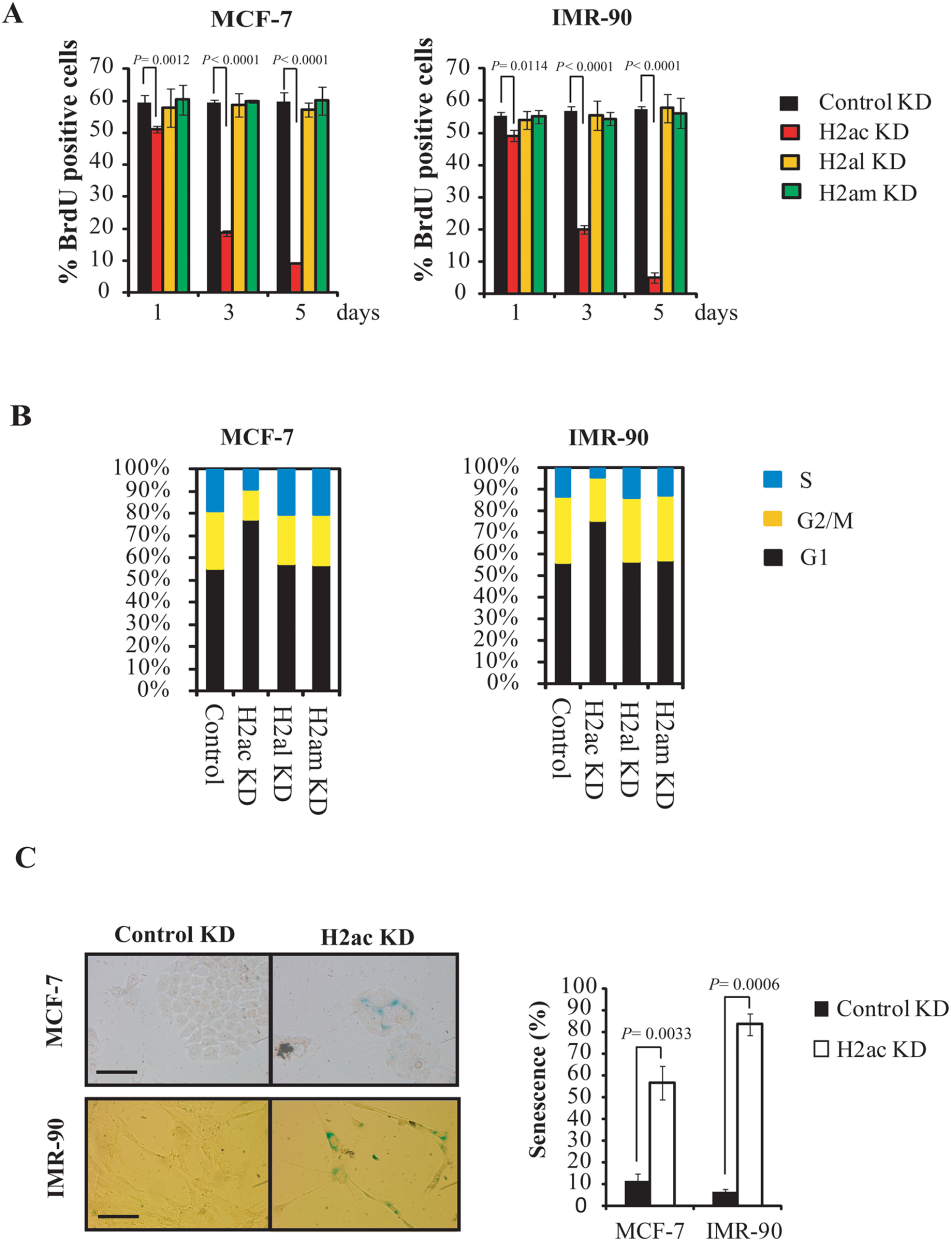
H2ac depletion induces cell arrest and cellular senescence. (A) BrdU incorporation assay of MCF-7 and IMR-90 cells, respectively with transfected H2ac-siRNA, H2al-siRNA, H2am-siRNA or control siRNA. 1, 3 and 5 days after H2ac depletion, cells were pulsed with BrdU for 4 hr. Then cells were stained with anti-BrdU antibody and percentage of BrdU the positive cells is indicated. The *P* value was calculated using a Student's two-tailed *t*-test. (B) Cell-cycle analysis of MCF-7 and IMR-90 cells, respectively with transfected H2ac-siRNA, H2al-siRNA, H2am-siRNA or control siRNA by using propidium iodide and flow cytometric analysis. (C) SA-β-gal staining was done for the analysis of cellular senescence of MCF-7 and IMR-90 cells transfected with control siRNAs or siH2ac for 5 days. Quantification of SA-β-gal-positive cells relative to total cells was obtained by counting 200 cells in three randomly chosen fields per dish. Three independent experiments were conducted. Data obtained from the representative experiment are shown. Bar represents the mean ± SD of three experiments. The *P* value was calculated using a Student's two-tailed *t*-test.

**Fig 7:**
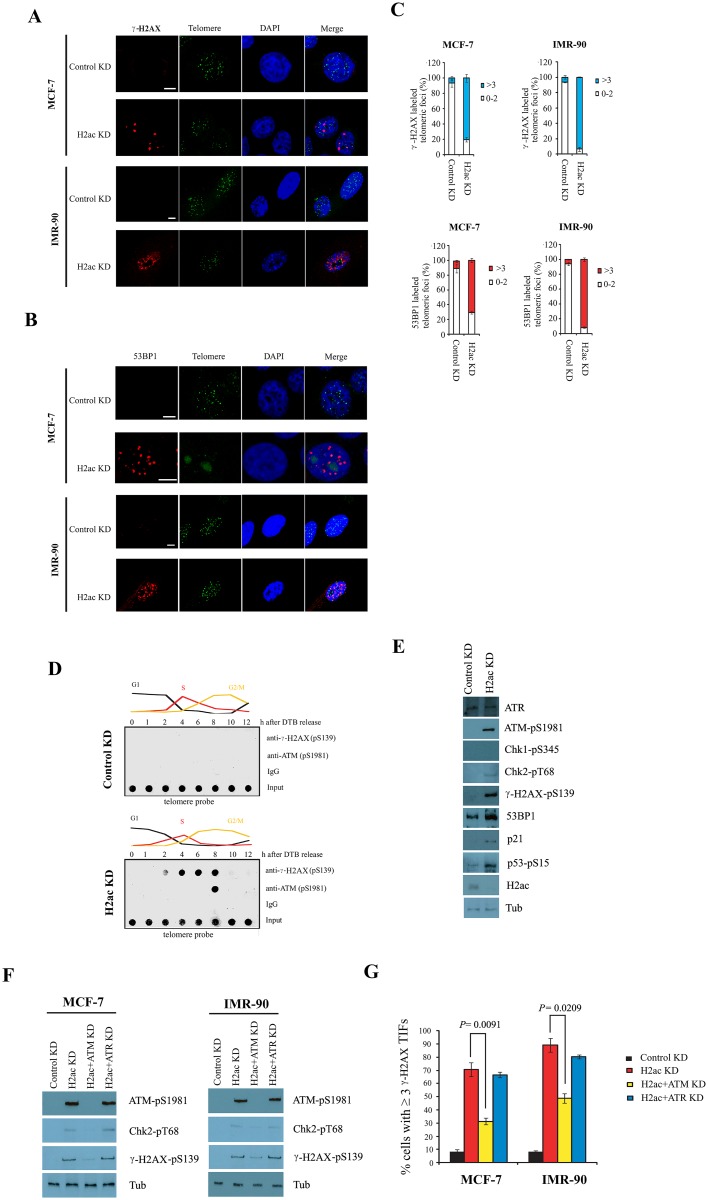
H2ac knockdown triggers an ATM-dependent DNA damage signal at telomeres. (A) Representative images of confocal sections of γ-H2AX foci (red) and telomere (green) in nuclei of MCF-7 and IMR-90 cells expressing either control KD or H2ac KD. The magnification of the images is shown as a scale bar (5 μm). (B) Representative images of confocal sections of 53BP1 foci (red) and telomere (green) in nuclei of MCF-7 and IMR-90 cells expressing either control knockdown (KD) or H2ac KD. The magnification of the images is shown as a scale bar (5 μm). (C) Quantitation of γ-H2AX-associated and 53BP1-associated telomeres in TIFs assays in cytogenetic preparations (mean ± SD; n = 3). (D) Using phosphorylated γ-H2AX (pS-139) and ATM (pS-1981) antibodies to perform telomere-ChIP analysis in MCF-7 extracts with transfected with control or H2ac siRNA, respectively, prepared at 0, 1, 2, 4, 6, 8, 10 and 12 hr after release from double-thymidine block. (E) Western blotting of cell extracts prepared from control KD, and H2ac KD cells using antibodies labeled on the right. (F) Western blotting of cell extracts prepared from control KD, H2ac KD, H2ac+ATM double KD and H2ac+ATR double KD cells using antibodies labeled on the right. (G) Percentage of cells containing three or more γ-H2AX-positive TIFs in control KD, H2ac KD, H2ac+ATM KD and H2ac+ATR KD cells (mean ± SD; n = 3; The *P* value was calculated using a Student's two-tailed *t*-test).

Telomere dysfunction can induce DNA damage response pathways, cell cycle arrest, apoptosis, and senescence [32–35]. Therefore, we examined whether the depletion of H2ac could also induce these cellular responses. Knockdown of H2ac for 1, 3 and 5 days resulted in inhibition of cellular DNA replication as measured by BrdU incorporation assay (Fig 6A). Flow cytometry analysis showed that the cell cycle was significantly arrested at the G1 phase three days after H2ac knockdown in both MCF-7 and IMR-90 cells (Fig 6B). In contrast, the depletion of other H2A histone isotypes, H2al and H2am, had no effect on DNA replication and cell cycle progression in both MCF-7 and IMR-90 cells (Fig 6A and 6B). Taken together, these data suggest that reduced levels of H2ac repressed cell proliferation. Furthermore, H2ac depleted cells expressed the senescence marker SA-β-galactosidase (Fig 6C), acquired a senescent morphology as well as induced the expression of senescence-associated p21 and S-15 phosphorylated p53 (Fig 7E).

Foci of phosphorylated γ-H2AX and 53BP1 (telomere dysfunction induced foci or TIFs), two markers for double-strand breaks (DSBs) [33,36,37], were strongly up-regulated in the H2ac depleted cells as compared with the control knockdown. The numbers of nuclei showing more than three phosphorylated γ-H2AX foci at telomeres was observed in 80% of H2ac depleted MCF-7 and in 93% of H2ac depleted IMR-90, a drastic increase as compared with the control knockdown (6.3% and 6.4% of the nuclei in MCF-7 and IMR-90, respectively, Fig 7A and 7C). Similarly, we also observed a significantly increased number of 53BP1 foci co-localized with telomeres in H2ac-depleted cells (70% and 91%of the nuclei with more than three foci/nucleus in MCF-7 and IMR-90, respectively) as compared to the control knockdown (9.6% and 5.7% of the nuclei with more than three foci/nucleus in MCF-7 and IMR-90, respectively, Fig 7B and 7C). Western blot analysis also showed that protein expression of phosphorylated γ-H2AX and 53BP1 were up-regulated in H2ac depleted cells as compared with control knockdown (Fig 7E). However, we also observed that many of the γ-H2AX /53BP1 foci in H2ac knockdown cells appeared to co-localize with non-telomeres. These data suggest that depletion of H2ac caused the accumulation of γ-H2AX /53BP1 foci not only at telomeric sequences with telomere dysfunction but also at non-telomeric sequences.

To study whether the H2ac depletion-induced DNA damage response was cell-cycle dependent, MCF-7 cells were synchronized at the G1/S boundary by double thymidine block [27]. Upon release from G1/S, cells were chased for the indicated times and were subjected to telomere-ChIP assay using an anti-γ-H2AX (pS139) probe. The telomere-ChIP analysis revealed that telomeres of H2ac-depleted cells became associated with phosphorylated γ-H2AX two hours after release from G1/S (Fig 7D). In contrast, no signal was observed throughout the cell cycle in control knockdown. This indicates that the cell cycle progression through mitosis is not required for the DNA damage response in H2ac-depleted cells.

It has been shown that depletion of TRF2 activates ATM through S1981 phosphorylation whereas removal of POT1 from telomeres initiates an ATR‐dependent DNA damage response (DDR) [33,38,39]. To determine which damage response pathway is involved in H2ac-depleted cells, we analyzed the expression of ATM and ATR using western blot. The result showed that ATM phosphorylated at S1981 and Chk2 phosphorylated at T68 were detected after H2ac knockdown but were absent in the control, whereas ATR and phosphorylated Chk1 at S345 were not found to be up-regulated after H2ac knockdown (Fig 7E). Furthermore, phosphorylated ATM, γ-H2AX and Chk2 were eliminated after ATM and H2ac double knockdown but persisted in ATR and H2ac double knockdown (Fig 7F). In contrast, ATR depletion had no effect on TIFs formation in H2ac-deficient cells, whereas the TIFs signal was significantly reduced in ATM and H2ac double knockdown cells (Fig 7G). Analysis of synchronized cells showed that activated ATM was observed in dysfunctional telomeres at the G2 phase in H2ac depleted cells, whereas no signal was detected throughout the cell cycle in the control knockdown (Fig 7D). These findings reveal that telomere dysfunction as a result of H2ac deficiency activates ATM, but not the ATR cell cycle checkpoint.

### XPF is essential for H2ac knockdown-mediated telomere deletion and 3’ G-strand overhang loss

**Fig 8:**
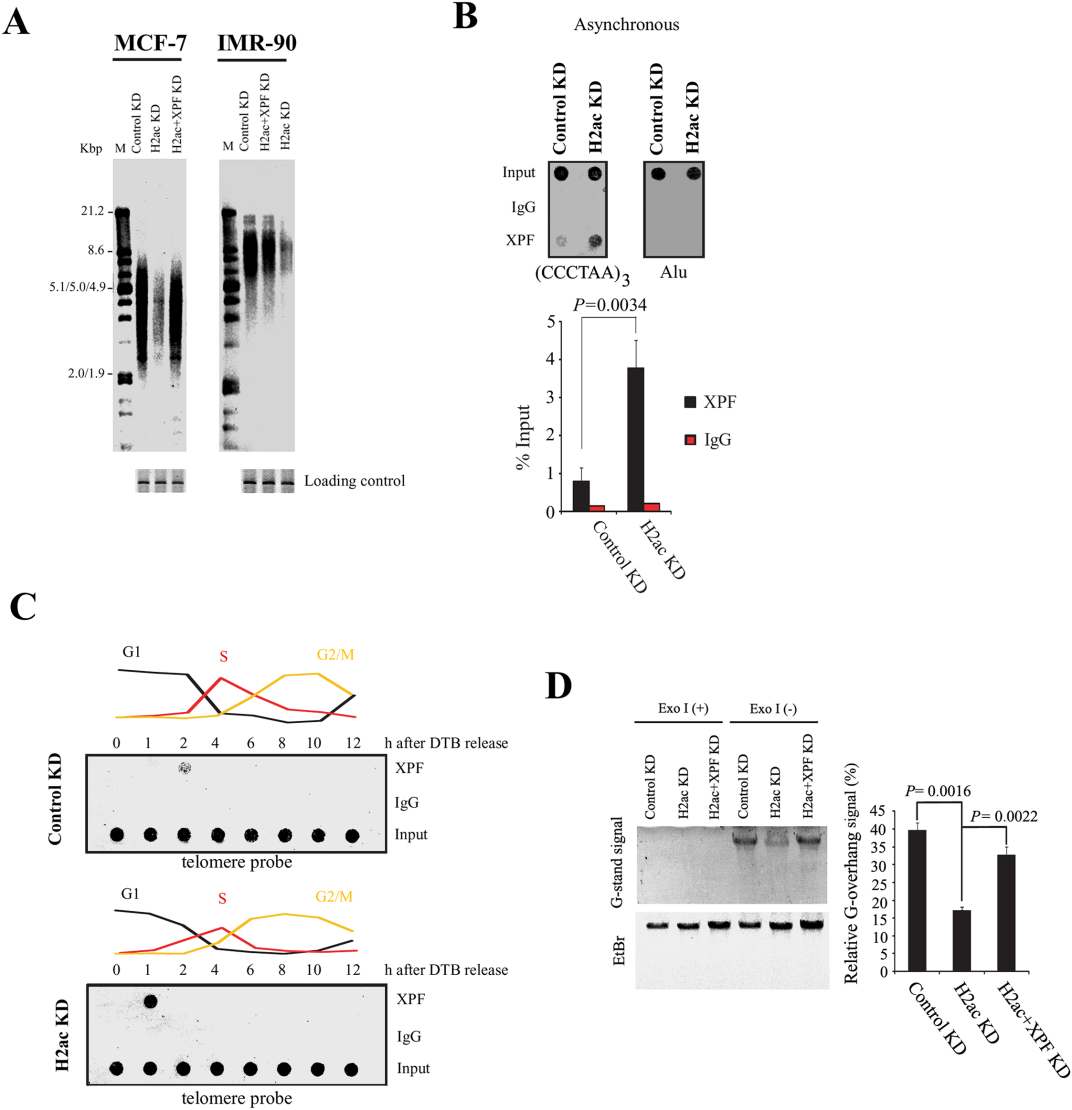
XPF is essential for H2ac depletion-mediated telomere deletion and loss of 3’ G-strand overhangs. (A) Knockdown of XPF rescued the telomere deletion induced by the treatment with H2ac-siRNA, as examined by TRF assay. (B) Telomere-ChIP assay showing the effect of H2ac depletion on the occupancy of XPF in telomeres with telomere-specific sequences or Alu sequences using dot blot. Quantification of telomeric-repeat DNA recovered in each ChIP is shown. Results are average of experiments performed in triplicate. The *P* value was calculated using a Student's two-tailed *t*-test. (C) Telomere-ChIP analysis MCF-7 extracts with transfected control siRNA and *H2ac*-siRNA, respectively, prepared 0, 1, 2, 4, 6, 8, 10 and 12 hr after release from double-thymidine block using XPF antibody. (D) Non-denaturing hybridization analysis of telomeric G-strand overhangs from control KD, H2ac KD and H2ac+XPF KD cells. Undigested genomic DNA was hybridized to DIG-labeled C-rich (CCCTAA)_3_ probe and then gel fractionated in 0.5× TBE. Relative amounts of overhangs were calculated by normalizing signals from non-denaturing gel (overhang signals) to the EtBr signal (representing total genomic DNA) and plotted. Results shown are representative of three independent experiments. Error bars represent one standard deviation. The *P* value was calculated using a Student's two-tailed *t*-test.

XPF was required for promoting loss of telomeric G-strand overhangs in TRF2 inhibited cells [40]. To evaluate the impact of XPF deficiency in cells depleted of H2ac, we carried out H2ac and XPF double knockdowns. Simultaneous knockdown of H2ac and XPF showed significantly less reduction of the telomeric repeat signal as determined by telomere restriction fragment analysis (Fig 8A). We further examined whether XPF was recruited to dysfunctional telomere in H2ac depleted cells by telomere-ChIP assay. Indeed, in asynchronous cells, an increased amount of XPF was recruited to telomeres in H2ac depleted cells as compared with control knockdown cells (Fig 8B). Interestingly, following double thymidine block release, the telomere-ChIP assay revealed that H2ac depletion resulted in the robust recruitment of XPF to the telomere at one hour after cells were released from double thymidine block at the G1/S transition. Whereas, only a weak signal was observed at two hours in synchronized cells of control knockdown released from double thymidine block at the G1/S transition (Fig 8C). These results suggest that the XPF is recruited to the telomere immediately after H2ac knockdown.

Given that XPF-ERCC1 activity has the potential to remove the 3’ G-strand overhang from telomeres after TRF2 inhibition [40] and H2ac depletion resulted in the disassociation of TRF2 as well as the recruitment of XPF with telomeres (Figs 4B and 8B), we examined the fate of 3’ G-strand overhangs in H2ac knockdown and simultaneous knockdown of H2ac and XPF by non-denaturing hybridization analysis to measure the relative changes in overhang abundance. Genomic DNA was first hybridized to a C-rich [(CCCTAA)_3_] probe under non-denaturing conditions to allow the probe to hybridize to the telomeric overhangs. Undigested whole genomic DNA was used in order to remain as a compact band that could concentrate the very low overhang signal into a small area in the gel for easy quantitative analysis. Relative signals from G-stranded overhangs hybridized to a telomere probe under native conditions were normalized to the total genomic DNA signal obtained after the DNA was stained by EtBr. DNA digested by exonuclease I (Exo I) to remove 3’ single-strand overhangs served as a negative control. As shown in Fig 8D, we detected a substantial decrease of G-strand specific signal intensities with H2ac-specific siRNA when compared with cells transfected with a siRNA control. This result indicates that XPF recruitment to the telomeres after H2ac knockdown results in the removal of 3’ G-strand overhangs.

### XPF is essential for the H2ac knockdown-mediated DNA damage response and genome abnormalities

**Fig 9:**
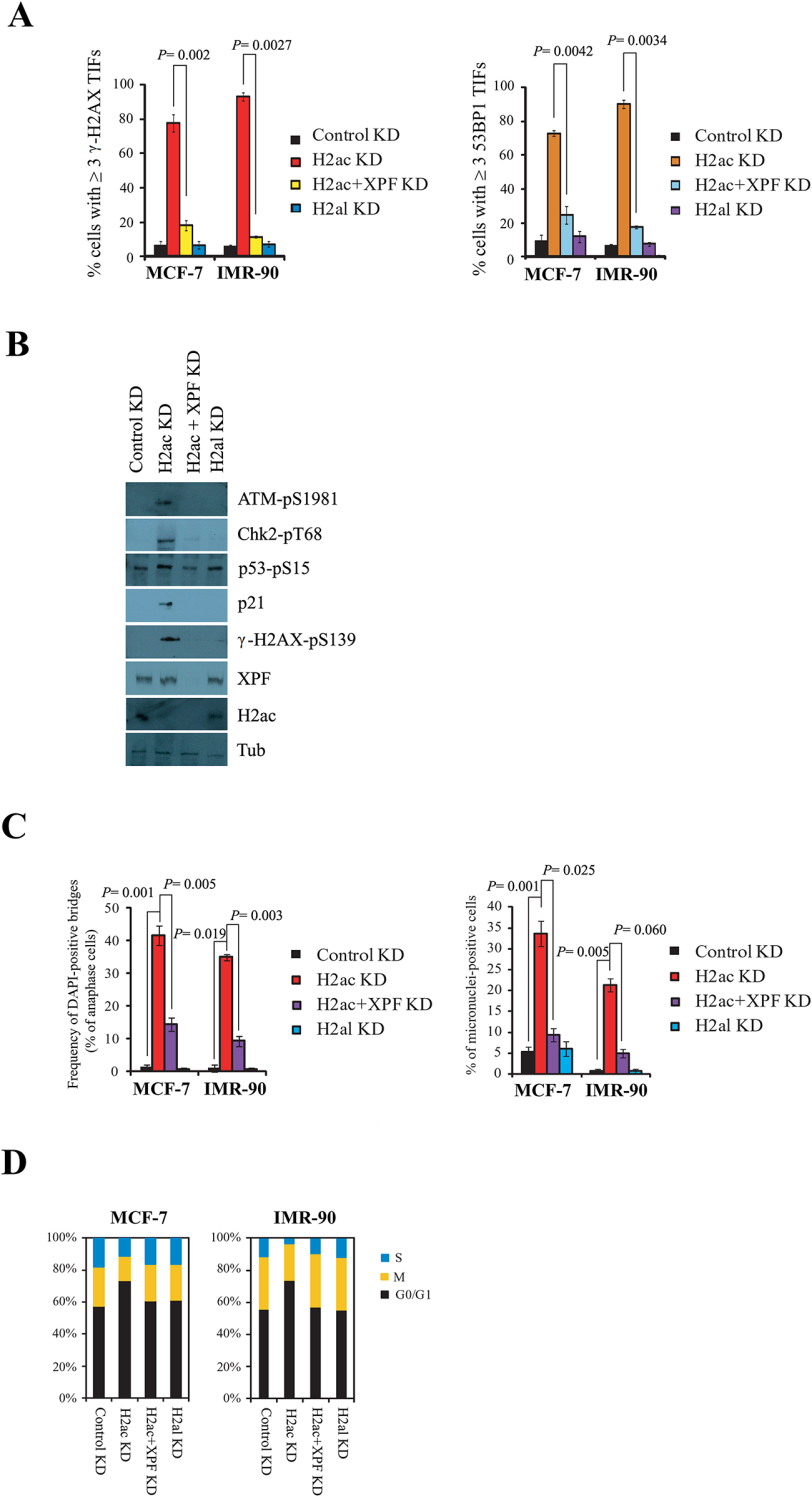
XPF is essential for the H2ac depletion-mediated DNA damage response and genome abnormalities. (A) Knockdown of XPF reduced the consequences of TIFs induced by the treatment with H2ac specific siRNA, as examined by telomere-FISH and immunofluorescence assay. The bar graph shows the mean number of TIFs per nucleus. The data are represented as mean ± SD. The *P* value was calculated using a Student's two-tailed *t*-test. (B) Western blotting of cell extracts prepared from control knockdown (KD), H2ac KD, H2ac+XPF double KD and H2al KD cells using antibodies labeled on the right. (C) The reduction of anaphase bridges and micronuclei in simultaneous knockdown of H2ac and XPF. Quantification of DAPI-positive anaphase bridges and micronuclei of cells treated with control, H2ac siRNA, or H2ac and XPF double siRNAs. 100 anaphase cells or interphase cells were analyzed. The *P* value was calculated using a Student's two-tailed *t*-test. (D) Knockdown of XPF reduced the cell arrested induced by the treatment with H2ac specific siRNA. Cell-cycle analysis of the indicated cells was assessed using propidium iodide staining and flow cytometry.

XPF-ERCC1 was required for the repair of DNA damage caused by uncapped telomeres through the activation of the homologous recombination (HR) and non-homologous end joining (NHEJ) pathways, leading to telomere length shortening and end to end fusions, respectively [41,42]. To explore the role of XPF-ERCC1 in the uncapped telomeres of DNA damage induced by H2ac depletion, the accumulation of phosphorylated γ-H2AX and 53BP1 after simultaneous knockdown of XPF and H2ac was investigated by telomere-PNA FISH and immunofluorescence. As expected, no significant differences were detected by observing the number of nuclei showing more than three TIFs at telomeres between control knockdown and double knockdown (Fig 9A). Consistently, western blot indicated no significant change between control and double knockdowns in DNA damage response factors, including activated ATM, Chk2 (pT68), p53 (pS-15), γ-H2AX (pS139) and p21 (Fig 9B) as compared with the induction of these signals in H2ac knockdown alone. Additionally, double knockdown also reduced the frequency of chromosome abnormalities compared with that found in H2ac single knockdown cells (Fig 9C).

In addition, flow cytometry analysis revealed that no significant differences in cell cycle progression were detected between the control knockdown and double knockdown (Fig 9D). These findings suggest that telomere dysfunction and DNA damage response induced by H2ac single knockdown is rescued by simultaneous knockdown of H2ac and XPF, and XPF is required for H2ac depletion induced damage signaling.

## Discussions

In this report we discovered an unexpected role of a histone H2A subtype, H2ac, as a regulator of telomere metabolism. First, we showed that showed that H2ac is critical in the maintenance of the telomere as depletion of this histone isotype resulted in loss of telomeric repeat sequences and induction of the DNA damage response as well as genome instability, cell cycle arrest and senescence. Interestingly, Sixteen replication-dependent histone H2A genes are distributed across at least three clusters; 12 are located in the Hist1 cluster on chromosome 6, three in the Hist2 cluster, and one in the Hist3 cluster on chromosome 1, which have been identified in the human genome [25]. These replication-dependent histone H2A isotypes differ in their coding sequences, but are similar in their amino acid sequences. Interestingly, H2ac differs from other canonical H2A histones in the Hist1 cluster at two positions in H2ac, the threonine at position 16 is changed to serine and the lysine residue at position 99 residue is changed to arginine residue in H2ac (S6 Fig). The consensus sequence varies in two positions for mouse H2A resulting in variation among the orthologous mouse histone forms [25]. It is not clear whether these variations have functional significance in relation to human H2ac. However, overexpression of H2ac mutants (S16A, S16T and R99K) and another H2A histone isotype, H2al, did not bring down TRF2 and POT1, indicating that two positions of H2ac were required for binding to these factors. Knockdown of several other H2A isotypes did not induce loss of telomeric repeat sequences, DNA damage response and genome instability; hence H2ac is specifically involved in these biological functions. Further studies are required to elucidate the important role that the two residues play in the regulation of telomere integrity, and whether they act as sites of post-translational modification.

Wang *et al*. showed that overexpression of TRF2^△B^ led to a loss of telomere-repeat signal intensity in restriction fragment-length assays [30]. However, expression of a POT1 mutant lacking the DNA binding domain or partial knockdown of POT1 with short hairpin RNA did not revealed a de-protection phenotype [43,44]. Interestingly, we found that the amino-terminal basic domain of TRF2 was necessary for the recruitment of H2ac to telomeres and H2ac-depleted cells had an accelerated loss of telomere-repeat signals and increased telomere signal-free ends similar to cells with the overexpressing TRF2^△B^ [30], suggesting that H2ac might cooperate with TRF2 in maintaining telomere repeats. In addition, loss of telomeric repeat DNA by H2ac depletion was observed with varying extents in telomerase-positive MCF-7 cells and telomerase-negative primary human lung fibroblast cells (IMR-90). Metaphase spreads showed that telomere signal free ends did not appear to affect all chromatids equally. Although some telomeres appeared to be lost completely, others were relatively unaffected. Further investigation is needed to understand the mechanism of selective chromosome abnormalities during H2ac depletion.

In this report, we showed that knockdown of H2ac activates a robust DDR at telomeres, suggesting that H2ac plays a crucial role in preventing telomere ends from being recognized as damaged DNA. These results are consistent with recent observations that the interaction of TRF2 with POT1 is essential for forming the t-loop complex with telomeric single-stranded DNA and protecting telomere end from DDR [45–48]. However, TRF2 and POT1 deletion act independently to activate two different DNA damage response pathways in mammalian cells. TRF2 has the ability to inhibit ATM autophosphorylation at S1981 to prevent its activation in response to DNA damage [49], whereas POT1 prevents activation of ATR through RPA exclusion [38,49,50]. Interestingly, we observed that H2ac depletion result in telomere fusion through activation of ATM, but not ATR. Given that ATR recruitment to DSB requires the formation of RPA-coated single stranded DNA [38,50], we also analyzed the accumulation of RPA following H2ac knockdown. Indeed, there was no RPA accumulation in the checkpoint response after H2ac knockdown (S7 Fig), consistent with the absence of the ATR response after H2ac knockdown. The absence of RPA accumulation is probably due to the loss of telomere overhangs through XPF after H2ac knockdown. Additionally, ATM depletion eliminated the accumulation of phosphorylated Chk2 and TIFs, and prevented the cell cycle arrest and senescence in H2ac deficient cells, indicating that the ATM checkpoint is required for H2ac-induced cell cycle arrest and senescence. ATM was also found to associate with the telomere in the G2 phase, potentially due to replication-associated damage [51]. Given that our data showed that TRF2 is disassociated from the telomere and spreads to nucleoplasm after H2ac-depletion, this would lead to ATM in a condition ready to be activated through autophosphorylation.

We also observed that many of the γ-H2AX/53BP1 foci do not actually appear to co-localize with telomeres. Because H2ac is the replication-dependent histone, it is possible that the suppression of H2ac leads to general genome-wide replication dysfunction. Indeed, in our BrdU incorporation assay, the data demonstrated that DNA replication was inhibited in H2ac knockdown. However, depletion of other H2A subtypes did not result in the arrest of DNA replication, suggesting that inhibition of DNA replication after H2ac knockdown is not due to the depletion of a H2A isotype per se but must result from the effect of the induction of the damage response pathway that inhibits the cell cycle.

Metaphase spreads showed that H2ac depletion did not affect affect all chromatids equally. This suggests that the consequences of telomere dysfunction did not result from DNA replication general defects after H2ac knockdown. Additionally, the telomere-ChIP assay showed that the association of γ-H2AX with telomere was observed at S phase and ATM was associated with the telomere at G2/M phase in H2ac-depleted cells. These data suggested that these DNA damage markers were specifically associated with a subset of telomeres in H2ac-depleted cells.

XPF knockdown would prevent the trimming of exposed single strand overhang in H2ac knockdown cells. However, the involvement of the XPF domain in the telomere maintenance remains elusive. After TRF2 inhibition, a partial loss of telomeric G-strand overhangs was observed that was dependent on ERCC1-XPF nuclease [40] and overexpression of TRF2 led to XPF-dependent telomere loss, increased DNA damage, aging and cancer in mouse keratinocytes [52]. Despite evidences indicating that ERCC1-XPF is required for telomere maintenance, Wu *et al*. demonstrated that the mutated XPF protein with mutations in its conserved nuclease domain was deficient in DNA repair, but could still function in TRF2-mediated telomere shortening [53]. Furthermore, considering only ~1% of ERCC1-XPF forms complexes with TRF2 [40,54], it is possible that XPF associates with telomeres through other complexes independent of TRF2. Recently, an Fanconi anemia (FA) protein, SLX4 (or BTBD12 or FANCP), that interacts with XPF-ERCC1, MUS81-EME1, and SLX1 was identified [55]. The complex was recruited to telomeres through TRF2 interacting with SLX4 independently of XPF, and functions as a “double-layer scaffold” to organize a multi-nuclease complex for regulating telomere length homeostasis mechanisms employed at long telomeres [56]. Thus, XPF could associate with telomeres by indirectly interacting with TRF2. Moreover, Wu *et al*. have previously shown that wild type XPF negatively regulates TRF2 association with telomere DNA [57]. Indeed, our data showed that TRF2 was reloaded onto telomeres in simultaneous knockdown of H2ac and XPF (S8 Fig) suggesting that the association of H2ac with TRF2 prevents telomere trimming by XPF recruitment. Thus, further studies of the involvement of the ERCC1-XPF complex in telomere trimming after H2ac knockdown will be needed to elucidate complex interplay with other overhang processing activities.

## Methods and Materials

### Cell culture and transfection

MCF-7 cells (ATCC^®^ HTB-22^™^) were grown in RPMI media 1640 medium supplemented with 10% fetal bovine serum. IMR90 cells (ATCC^®^ CCL-186^™^) were cultured in Eagle's Minimum Essential Medium (EMEM) with 10% fetal bovine serum. HEK-293T cells (ATCC^®^ CRL-3216^™^) were maintained in Dulbecco’s Modified Eagle Medium (DMEM) supplemented with L-glutamine, penicillin/streptomycin, nonessential amino acids and 10% fetal bovine serum. Cells were transfected using LiopfectAMINETM RNAiMAX and Lipofectamine^®^ LTX with Plus^™^ Reagent (Invitrogen) according to the instructions of the manufacturer. The siRNAs were obtained from Sigma-Aldrich.

### Transfection and immunoprecipitation

HEK-293T or MCF-7 cells were transiently transfected by the liposome method, and whole cell extracts were prepared 36–48 hours post-transfection. The extracts were incubated with anti-HA agarose (Sigma), and anti-hist1H2AC (H00008334-M01, Novus Biologicals) at 4°C for 2–4 hours. Immunoprecipitated proteins were resolved by SDS-PAGE, transferred onto PVDF membranes and probed using various antibodies.

### *In vitro* pull down assay

FLAG-, MYC- and HA-tagged proteins were created using an EasyXpress Protein Synthesis kit (QIAGEN) according to the manufacturer’s protocol. Briefly, *in vitro*-translated proteins were incubated with 10 mL EZview^™^ anti-HA affinity gel (Sigma, St. Louis, MO) overnight at 4°C. The beads were retrieved and washed five times in 500 μL of PBS containing 0.5% NP-40 and boiled in 20 uL 2× sample buffer (250 mM Tris-HCL, 8% SDS, 40% glycerol, 0.04% bromophenol blue, and 400 mM dithiothreitol). The pulled down proteins were analyzed by immunoblotting.

### Chromatin Immunoprecipitation (ChIP)

The ChIP assays were performed according to the manufacturer’s protocol (Upstate Biotechnology, Inc., Lake Placid, NY), with the exception of the conditions for sonication that were changed to five times for 30 seconds each at 10% output. The antibodies used for the immunoprecipitations were anti-TRF2 (ab13579, abcam), anti-TRF1 (ab1423, abcam), anti-POT1 (ab21382, abcam), anti-HA (ab9110, abcam), anti-hist1H2AC (Novus Biologicals), anti-Histone H3.3 (ab62642, abcam) anti-γ-H2AX (pS-139) (ab2893, abcam), anti-ATM (pS-1981) (ab36810, abcam) and anti-XPF (ab17798, abcam). Non-specific rabbit polyclonal antibodies were used as a negative control. After reversing the protein-DNA cross-linking of the immunoprecipitated complexes, DNA was extracted for dot blotting analysis.

### Dot blot analysis

For dot blot analysis, ChIP DNA was denatured at 95°C and dot blotted on hybond membrane (Amersham) in 2X SSC buffer. Membranes were pre-hybridized in Rapid-Hyb buffer (Amersham) for 15 min. Following this, hybridization with a DIG-labelled telomeric probe (CCCTAA)_3_, DIG-labeled Alu probe (5'—GGCCGGGCGCGGTGGCTCACGCCTGTAATCCCAGCA -3') and DIG-labeled α-satellite repeat sequences probe (5’-AGAGTGTTTCAAAACTGCTCTATCA AAAGGAATGTTCAACGCGTGATC-3’) was performed for 3 hours at 42°C and membranes washed with 2X SSC and 0.1% SDS three times before exposing overnight on phosphoimager imaging plate. All data were scanned using FUJI Phosphoimager FLA2000. Data was processed and quantified using Multi Gauge image analysis software.

### ChIP sequencing

After cross-linking, chromatin was sonicated to reduce the size of DNA. The sequencing libraries were constructed from immunoprecipitated and input DNA using TruSeq ChIP Sample Preparation Kit (Illumina Inc., USA) according to the manufacturer’s instruction. The adapter-ligated DNA library was size-selected (300–400 bp) on a 2% agarose gel and amplified by PCR for 16–18 cycles using KAPA HiFi DNA Polymerase (Kapa Biosystems). Single-end DNA sequencing was performed on HiSeq2000 (Illumina Inc., USA) located at National Yang-Ming University VYM Sequencing Core Facility of Genome Research Center.

### ChIP-seq data analysis

The quality of NGS short reads was evaluated using FastQC (v0.10.1), followed by mapping of raw reads to the human reference genome (assembly hg18) using bowtie2 (v2.0.6) [58]. Duplicates were removed with the MarkDuplicates program from software suite Picard (v1.92). A total of 7.0, 5.7, 6.2, 6.7, 16.1 and 24.8 million uniquely aligned reads were obtained for the overexpressed HA-H2ac and HA (control) duplicates, and inputs for the two conditions, respectively. The predominant insert-size (fragment length) was estimated using the phantompeakqualtools package [59]. The Irreproducible Discovery Rate (IDR) framework and MACS (v2.0.10) were used for peak calling from replicate experiments [59,60]. The findMotifsGenome.pl program from software suite HOMER was used to perform de novo motif discovery using the peaks identified from MACS as input [61].

### Telomeric motif analysis

The fuzznuc tool from the EMBOSS suite (v6.5.7) was used to search for the occurrence of telomeric pattern TTAGGG (and its complementary pattern CCCTAA) in the FASTQ files, with zero mismatch allowed. The total number of neighboring telomeric patterns occurring on the same strand in each reads was tabulated.

### Accession codes

The ChIP-Seq data have been deposited to ArrayExpress under accession number E-MTAB-3160.

### Flow cytometric analysis

For flow cytometric analysis, cells were collected by trypsinization, centrifuged and resuspended in PBS, and the fixed by adding methanol to 90% at -20°C. Then fixed cells were washed with PBS and resupended in 4 mM sodium citrate containing 30U/ml RNAase A, 0.1% Triton X-100 and 50g μg/ml propidium iodide and incubated 10 min at 37°C. Data were collected using a FACScan apparatus.

### Telomeric single-strand assay

The non-denaturing hybridization assay to detect the telomeric single G strand was carried out as described previously with some modifications [62,63]. Briefly, Five micrograms of DNA were incubated overnight at 50°C with 1 pmol of DIG-labelled telomeric C-rich (CCCTAA)_3_ probe (Sigma-Aldrich) in hybridization buffer (50 mM NaCl, 10 mM Tris HCl at pH 8, 1 mM EDTA at pH 8.0). DNA that had been digested with 10 U of exonuclease I (20 U/mL) (life technologies) in a 20 μl reaction for 1 h at 37°C and terminated by adding EDTA served as a negative control. The free probe was separated from bulk DNA by electrophoresis on a 0.8% agarose gel containing 0.01% ethidium bromide for 3 h at 45 V. The gel was dried at 50°C, washed in water, dried again a few minutes, and probe detection followed the protocol of the manufacturer of the DIG High Prime DNA Labeling and Detection Starter Kit II (Roche), quantified, and normalized with regard to total DNA in that sample given by the ethidium bromide signals.

### Indirect immunofluorescence

Cells cultured in 4-well Culture SlideChambers (BD Biosciences) were fixed for 10 min at room temperature with 4% paraformaldehyde and rinsed three times with PBS. The fixed samples incubated for 10 min with PBS containing 0.25% Triton X-100 and rinsed three times with PBS. Samples treated with 1% BSA in PBST for 30 min to block unspecific binding of the antibodies following the mixture of two primary antibodies in 1% BSA in PBST overnight at 4°C, then rinsed, and incubated with secondary antibody. Nuclei were stained with DAPI.

### Telomere FISH staining of metaphase spreads

Briefly, the cultures were treated with 100 ng/ml colcemid for 4–6 hours. Cells were hypotonically swollen in 75 mM KCl at 37°C for 15–30 minutes. Cells were fixed by adding ice-cold fixative (1:3 acetic acid: methanol). Metaphase spreads were obtained by dropping fixed cells onto slides. To dehydrate the slides, we used a graded ethanol series (70% for 3 min, 90% for 2 min and 100% for 2 min) and air-dried. The dehydrated slides were overlaid with 0.3 μg/ml Alexa 488–OO-(CCCTAA)_3_ PNA probes (Panagene) in PNA hybridization solution (70% deionized formamide, 0.25% NEN blocking reagent (PerkinElmer), 10 mM Tris (pH 7.5), 4 mM Na2HPO4, 0.5 mM citric acid and 1.25 mM MgCl2), incubated at 80°C for 3 min and hybridized at room temperature for 2 hours. The slides were washed in PNA wash A (70% formamide and 10 mM Tris (pH 7.5)), then washed in PNA wash B (50 mM Tris (pH 7.5), 150 mM NaCl and 0.08% Tween-20) and added DAPI at 50 ng/ml in the final wash. The slides were rinsed in deionized water and mounted them in DABCO (2.3% 1, 4-diazabicyclo (2.2.2) octane (Sigma), 90% glycerol and 50 mM Tris (pH 8.0)).

### Statistical analysis

All experiments were repeated between 3 to 5 times and the data are expressed as average ± SD. Statistical analysis of the data was conducted by pairwise Student's *t* test.

## Supporting Information

S1 Fig Knockdown of H2ac eliminated ChIP of telomeric DNA. Telomere-ChIP assays using anti-H2ac antibody were performed in MCF-7 and IMR-90 cells treated with control siRNA (black), H2ac siRNA#1(red) and siRNA#2 (green) using telomere-specific sequences or Alu sequences as control. Quantification of TTAGGG repeat DNA recovered in each ChIP is shown below. Results are average of experiments performed in triplicates.
(DOCX)

S2 Fig The specificity of antibodies was determined by competitive western blotting. The raw data of western blotting using anti-H2ac, anti-TRF2 and anti-POT1 with or without H2ac (Abnova, H00008334-P01), TRF2 (abcam, ab152737) and POT1 (OriGene, TP316275) proteins served as competitors. Tubulin used as internal controls.
(DOCX)

S3 Fig Analysis of the specificity of H2ac siRNA. (**A**) Alignment sequences of two H2ac scrambled siRNA and H2ac specific siRNA. (**B**) Expression levels of *H2ac* in MCF-7 cells with two H2ac scrambled siRNA or H2ac specific siRNA. mRNA expression levels were determined by quantitative RT-PCR and normalized against 18S rRNA. (**C**) MCF-7 cells were harvested at day 5 after three separate transfections with two H2ac scrambled siRNA and H2ac specific siRNA. Telomere-repeat length and intensity was measured by restriction digest of genomic DNA with AluI/MboI and Southern hybridization with DIG-labeled (TTAGGG)_4_ probe (top panel). The G3PDH region was used as a control for DNA loading (bottom panel). The position of MWs (kb) is indicated on the left.
(DOCX)

S4 Fig TRF assay in MCF-7 treated with H2al or H2am siRNA. MCF-7 cells were harvested at day 5 after three separate transfections with control, H2al and H2am siRNAs. Telomere-repeat length and intensity was measured by restriction digest of genomic DNA with HinfI/RsaI and Southern hybridization with DIG-labeled (TTAGGG)_4_ probe (top panel). The G3PDH region was used as a control for DNA loading (bottom panel). The position of MWs (kb) is indicated on the left.
(DOCX)

S5 Fig MN-γ-H2AX (+)-telomere (+) in H2ac depleted cells. Cells with H2ac siRNA were grown on coverslips in 6-well plates before they were processed for telomere FISH and immunofluorescence staining with anti γ–H2AX antibody. Scale bar, 5 μm.
(DOCX)

S6 Fig H2ac and canonical H2A share a common amino acid change. Protein sequence alignment of H2A and H2ac. Positions of divergence are highlighted in red.
(DOCX)

S7 Fig No RPA accumulation at telomere in H2ac-depleted cells. Telomere-ChIP assays using anti-RPA 70 and anti-RPA 32 antibodies were performed in MCF-7 treated with control or H2ac siRNAs followed by dot blotting using telomere-specific sequences or Alu sequences as control.
(DOCX)

S8 Fig Simultaneous knockdown of H2ac and XPF result in the reloading of TRF2 onto telomeres. Telomere-ChIP assay showing the effect of simultaneously depletion of H2ac and XPF on the occupancy of TRF2 in telomeres with telomere-specific sequences or Alu sequences using dot blot. Quantification of telomeric-repeat DNA recovered in each ChIP is shown. Results are average of experiments performed in triplicate. The *P* value was calculated using a Student's two-tailed *t*-test.
(DOCX)
