# Supplementary material for: An H2A Histone Isotype, H2ac, Associates with Telomere and Maintains Telomere Integrity
Source: PLoS One. 2016 May 26;11(5):e0156378. doi: 10.1371/journal.pone.0156378 (PMC4882029; doi:10.1371/journal.pone.0156378)
Supplement: S1 Fig — Telomere-ChIP assays using anti-H2ac antibody were performed in MCF-7 and IMR-90 cells treated with control siRNA (black), H2ac siRNA#1(red) and siRNA#2 (green) using telomere-specific sequences or Alu sequences as control. Quantification of TTAGGG repeat DNA recovered in each ChIP is shown below. Results are average of experiments performed in triplicates. (DOCX) [file pone.0156378.s001.docx]

**S1 Fig**


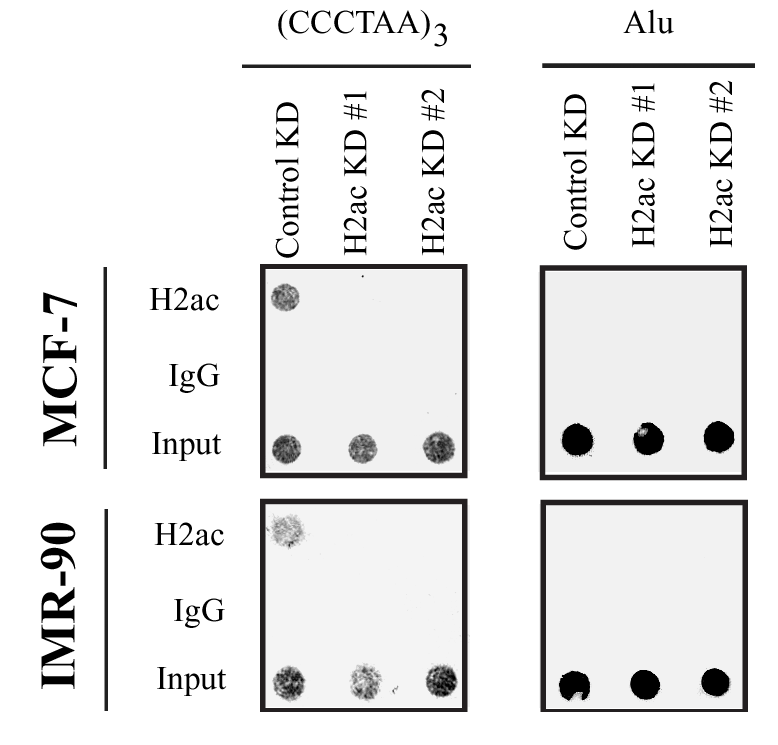


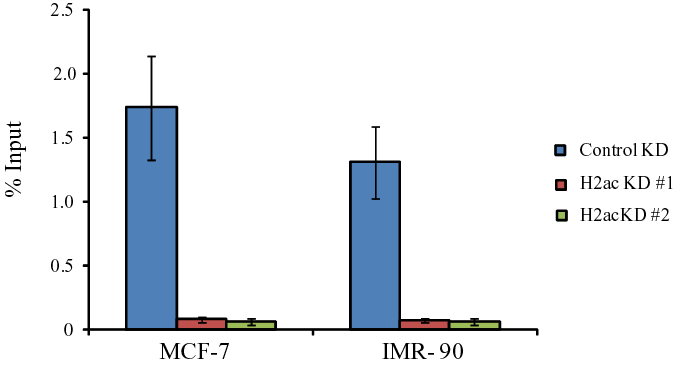


**S1 Fig.** **Knockdown of H2ac eliminated ChIP of telomeric DNA**. Telomere-ChIP assays using anti-H2ac antibody were performed in MCF-7 and IMR-90 cells treated with control siRNA (black), H2ac siRNA#1(red) and siRNA#2 (green) using telomere-specific sequences or Alu sequences as control. Quantification of TTAGGG repeat DNA recovered in each ChIP is shown below. Results are average of experiments performed in triplicates
