# Supplementary material for: An H2A Histone Isotype, H2ac, Associates with Telomere and Maintains Telomere Integrity
Source: PLoS One. 2016 May 26;11(5):e0156378. doi: 10.1371/journal.pone.0156378 (PMC4882029; doi:10.1371/journal.pone.0156378)
Supplement: S2 Fig — The raw data of western blotting using anti-H2ac, anti-TRF2 and anti-POT1 with or without H2ac (Abnova, H00008334-P01), TRF2 (abcam, ab152737) and POT1 (OriGene, TP316275) proteins served as competitors. Tubulin used as internal controls. (DOCX) [file pone.0156378.s002.docx]

**S2 Fig**


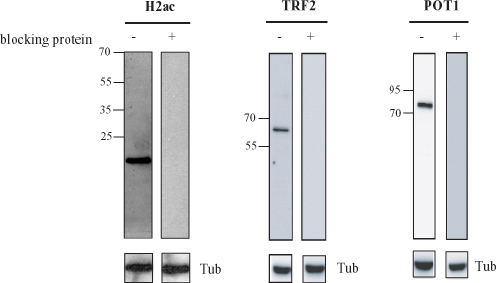


**S2 Fig. The specificity of antibodies was determined by competitive western blotting.** The raw data of western blotting using anti-H2ac, anti-TRF2 and anti-POT1 with or without H2ac (Abnova, H00008334-P01), TRF2 (abcam, ab152737) and POT1 (OriGene, TP316275) proteins served as competitors. Tubulin used as internal controls.
