# Supplementary material for: An H2A Histone Isotype, H2ac, Associates with Telomere and Maintains Telomere Integrity
Source: PLoS One. 2016 May 26;11(5):e0156378. doi: 10.1371/journal.pone.0156378 (PMC4882029; doi:10.1371/journal.pone.0156378)
Supplement: S3 Fig — (A) Alignment sequences of two H2ac scrambled siRNA and H2ac specific siRNA. (B) Expression levels of H2ac in MCF-7 cells with two H2ac scrambled siRNA or H2ac specific siRNA. mRNA expression levels were determined by quantitative RT-PCR and normalized against 18S rRNA. (C) MCF-7 cells were harvested at day 5 after three separate transfections with two H2ac scrambled siRNA and H2ac specific siRNA. Telomere-repeat length and intensity was measured by restriction digest of genomic DNA with AluI/MboI and Southern hybridization with DIG-labeled (TTAGGG)4 probe (top panel). The G3PDH region was used as a control for DNA loading (bottom panel). The position of MWs (kb) is indicated on the left. (DOCX) [file pone.0156378.s003.docx]

**S3 Fig**


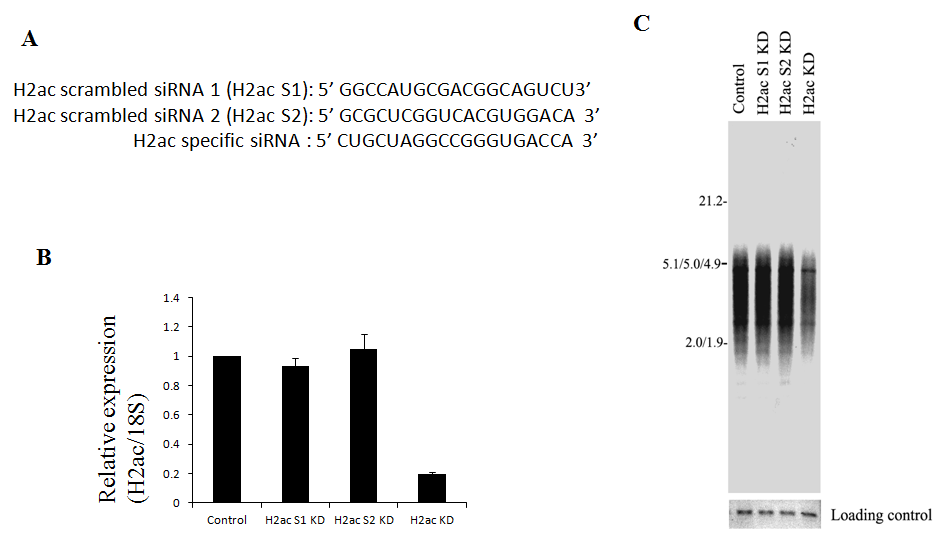


**S3 Fig. Analysis of the specificity of H2ac siRNA.** (**A**) Alignment sequences of two H2ac scrambled siRNA and H2ac specific siRNA. (**B**) Expression levels of *H2ac* in MCF-7 cells with two H2ac scrambled siRNA or H2ac specific siRNA. mRNA expression levels were determined by quantitative RT-PCR and normalized against 18S rRNA. (**C**) MCF-7 cells were harvested at day 5 after three separate transfections with two H2ac scrambled siRNA and H2ac specific siRNA. Telomere-repeat length and intensity was measured by restriction digest of genomic DNA with AluI/MboI and Southern hybridization with DIG-labeled (TTAGGG)_4_ probe (top panel). The G3PDH region was used as a control for DNA loading (bottom panel). The position of MWs (kb) is indicated on the left.
