# Supplementary material for: An H2A Histone Isotype, H2ac, Associates with Telomere and Maintains Telomere Integrity
Source: PLoS One. 2016 May 26;11(5):e0156378. doi: 10.1371/journal.pone.0156378 (PMC4882029; doi:10.1371/journal.pone.0156378)
Supplement: S4 Fig — MCF-7 cells were harvested at day 5 after three separate transfections with control, H2al and H2am siRNAs. Telomere-repeat length and intensity was measured by restriction digest of genomic DNA with HinfI/RsaI and Southern hybridization with DIG-labeled (TTAGGG)4 probe (top panel). The G3PDH region was used as a control for DNA loading (bottom panel). The position of MWs (kb) is indicated on the left. (DOCX) [file pone.0156378.s004.docx]

**S4 Fig**


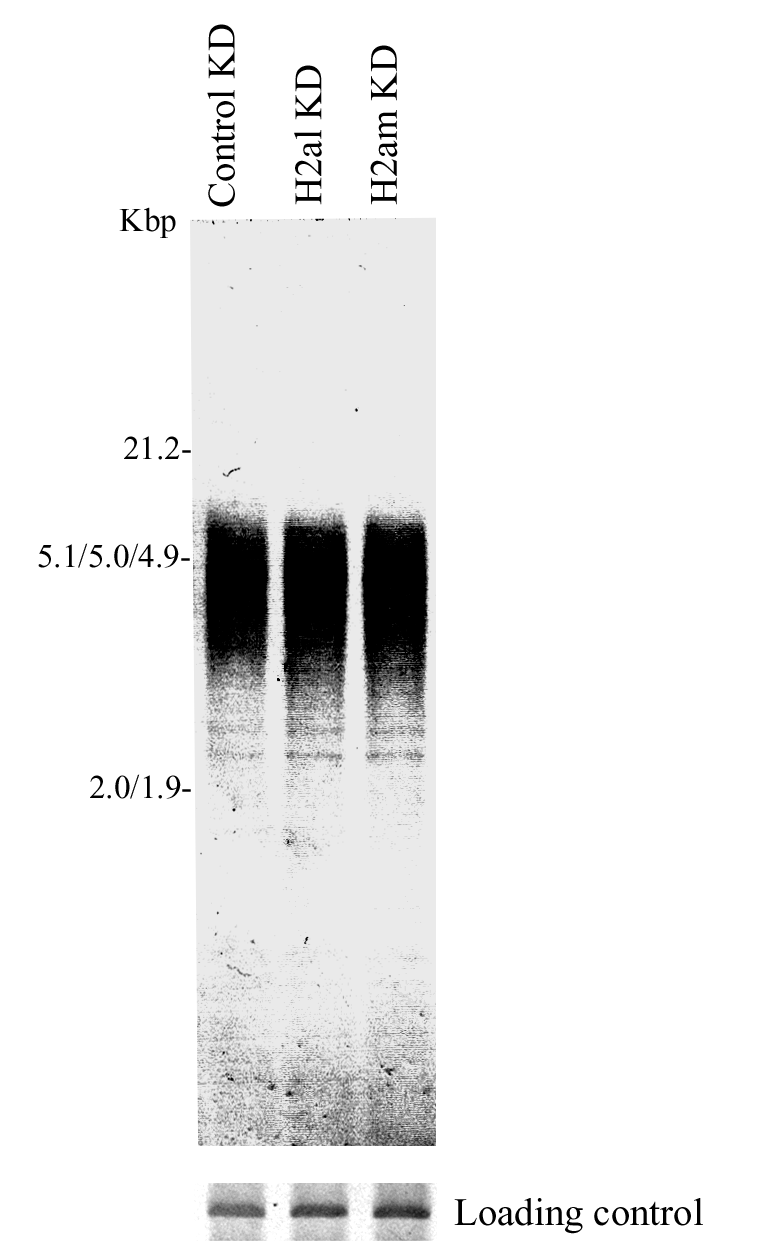


**S4 Fig. TRF assay in MCF-7 treated with H2al or H2am siRNA.** MCF-7 cells were harvested at day 5 after three separate transfections with control, H2al and H2am siRNAs. Telomere-repeat length and intensity was measured by restriction digest of genomic DNA with HinfI/RsaI and Southern hybridization with DIG-labeled (TTAGGG)_4_ probe (top panel). The G3PDH region was used as a control for DNA loading (bottom panel). The position of MWs (kb) is indicated on the left.
