# Supplementary material for: An H2A Histone Isotype, H2ac, Associates with Telomere and Maintains Telomere Integrity
Source: PLoS One. 2016 May 26;11(5):e0156378. doi: 10.1371/journal.pone.0156378 (PMC4882029; doi:10.1371/journal.pone.0156378)
Supplement: S5 Fig — Cells with H2ac siRNA were grown on coverslips in 6-well plates before they were processed for telomere FISH and immunofluorescence staining with anti γ–H2AX antibody. Scale bar, 5 μm. (DOCX) [file pone.0156378.s005.docx]

**S5 Fig**


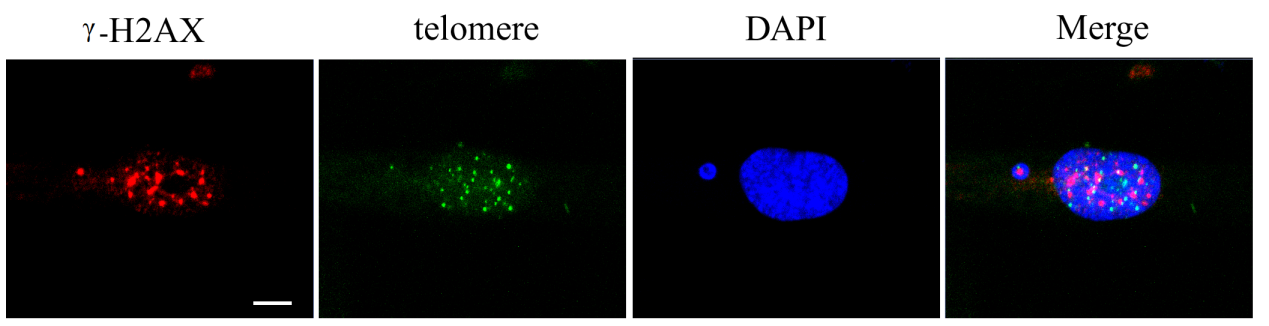


**S5 Fig. MN-γ-H2AX (+)-telomere (+) in H2ac depleted cells.** Cells with H2ac siRNA were grown on coverslips in 6-well plates before they were processed for telomere FISH and immunofluorescence staining with anti γ–H2AX antibody. Scale bar, 5 μm.
