# Supplementary material for: An H2A Histone Isotype, H2ac, Associates with Telomere and Maintains Telomere Integrity
Source: PLoS One. 2016 May 26;11(5):e0156378. doi: 10.1371/journal.pone.0156378 (PMC4882029; doi:10.1371/journal.pone.0156378)
Supplement: S6 Fig — Protein sequence alignment of H2A and H2ac. Positions of divergence are highlighted in red. (DOCX) [file pone.0156378.s006.docx]

**S6 Fig**


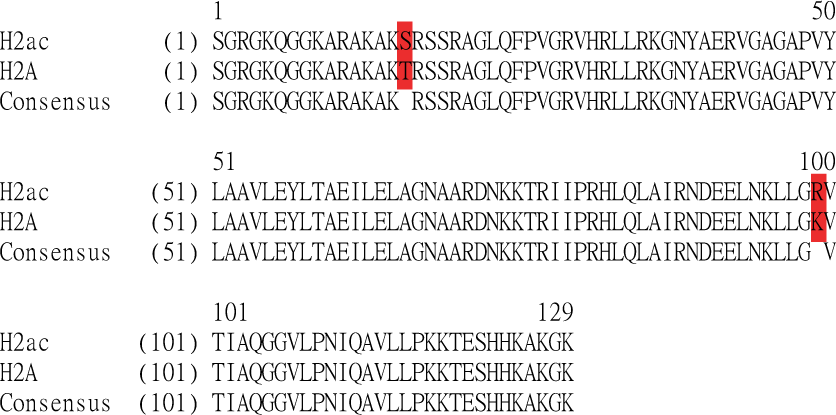


**S6 Fig. H2ac and canonical H2A share a common amino acid change.** Protein sequence alignment of H2A and H2ac. Positions of divergence are highlighted in red.
