# Supplementary material for: An H2A Histone Isotype, H2ac, Associates with Telomere and Maintains Telomere Integrity
Source: PLoS One. 2016 May 26;11(5):e0156378. doi: 10.1371/journal.pone.0156378 (PMC4882029; doi:10.1371/journal.pone.0156378)
Supplement: S7 Fig — Telomere-ChIP assays using anti-RPA 70 and anti-RPA 32 antibodies were performed in MCF-7 treated with control or H2ac siRNAs followed by dot blotting using telomere-specific sequences or Alu sequences as control. (DOCX) [file pone.0156378.s007.docx]

**S7 Fig**


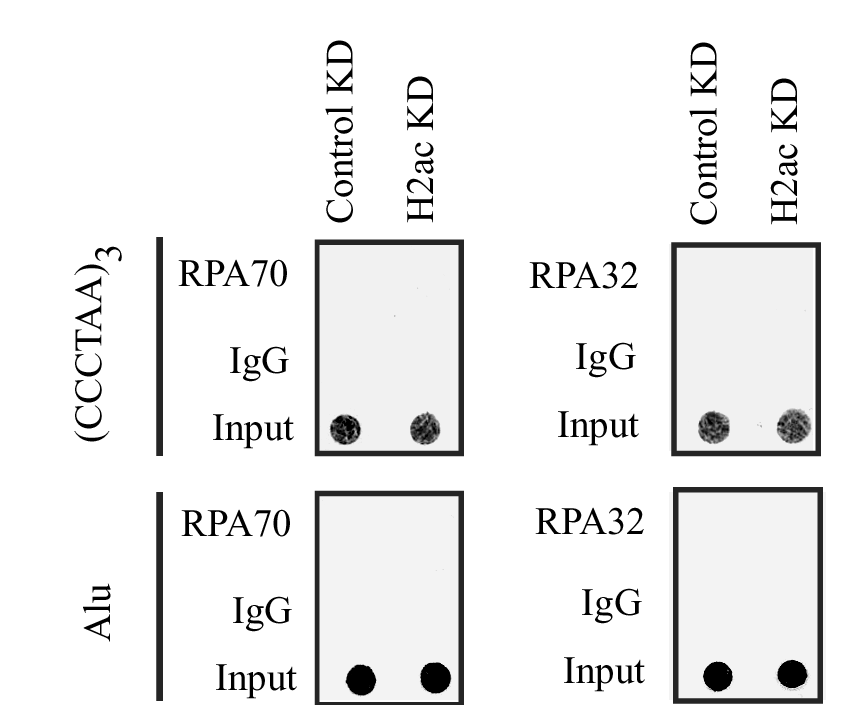


**S7 Fig. No RPA accumulation at telomere in H2ac-depleted cells.** Telomere-ChIP assays using anti-RPA 70 and anti-RPA 32 antibodies were performed in MCF-7 treated with control or H2ac siRNAs followed by dot blotting using telomere-specific sequences or Alu sequences as control.
