# Supplementary material for: An H2A Histone Isotype, H2ac, Associates with Telomere and Maintains Telomere Integrity
Source: PLoS One. 2016 May 26;11(5):e0156378. doi: 10.1371/journal.pone.0156378 (PMC4882029; doi:10.1371/journal.pone.0156378)
Supplement: S8 Fig — Telomere-ChIP assay showing the effect of simultaneously depletion of H2ac and XPF on the occupancy of TRF2 in telomeres with telomere-specific sequences or Alu sequences using dot blot. Quantification of telomeric-repeat DNA recovered in each ChIP is shown. Results are average of experiments performed in triplicate. The P value was calculated using a Student's two-tailed t-test. (DOCX) [file pone.0156378.s008.docx]

**S8 Fig**


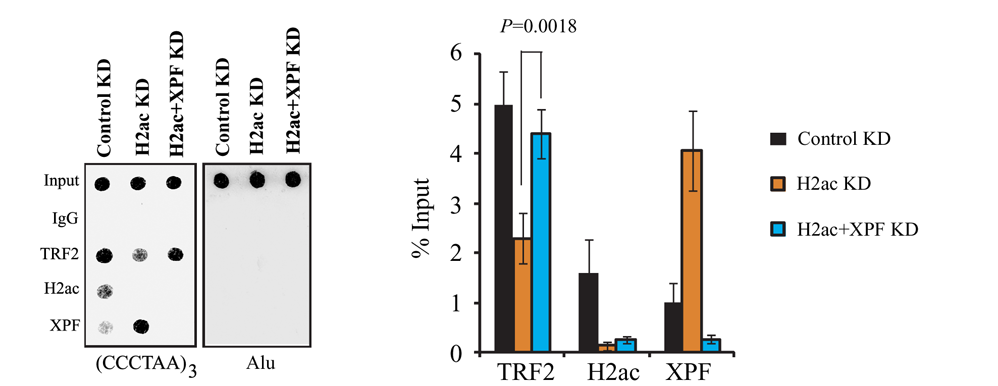


**S8 Fig. Simultaneous knockdown of H2ac and XPF result in the reloading of TRF2 onto telomeres.** Telomere-ChIP assay showing the effect of simultaneously depletion of H2ac and XPF on the occupancy of TRF2 in telomeres with telomere-specific sequences or Alu sequences using dot blot. Quantification of telomeric-repeat DNA recovered in each ChIP is shown. Results are average of experiments performed in triplicate. The *P* value was calculated using a Student's two-tailed *t*-test.
